# Lso2 is a conserved ribosome-bound protein required for translational recovery in yeast

**DOI:** 10.1371/journal.pbio.2005903

**Published:** 2018-09-12

**Authors:** Yinuo J. Wang, Pavanapuresan P. Vaidyanathan, Maria F. Rojas-Duran, Namrata D. Udeshi, Kristen M. Bartoli, Steven A. Carr, Wendy V. Gilbert

**Affiliations:** 1 Department of Molecular Biophysics and Biochemistry, Yale University, New Haven, Connecticut, United States of America; 2 Department of Biology, Massachusetts Institute of Technology, Cambridge, Massachusetts, United States of America; 3 Microbiology Graduate Program, Massachusetts Institute of Technology, Cambridge, Massachusetts, United States of America; 4 The Broad Institute of MIT and Harvard, Cambridge, Massachusetts, United States of America; Case Western Reserve University, United States of America

## Abstract

Ribosome-binding proteins function broadly in protein synthesis, gene regulation, and cellular homeostasis, but the complete complement of functional ribosome-bound proteins remains unknown. Using quantitative mass spectrometry, we identified late-annotated short open reading frame 2 (Lso2) as a ribosome-associated protein that is broadly conserved in eukaryotes. Genome-wide crosslinking and immunoprecipitation of Lso2 and its human ortholog coiled-coil domain containing 124 (CCDC124) recovered 25S ribosomal RNA in a region near the A site that overlaps the GTPase activation center. Consistent with this location, Lso2 also crosslinked to most tRNAs. Ribosome profiling of yeast lacking *LSO2* (*lso2Δ*) revealed global translation defects during recovery from stationary phase with translation of most genes reduced more than 4-fold. Ribosomes accumulated at start codons, were depleted from stop codons, and showed codon-specific changes in occupancy in *lso2Δ*. These defects, and the conservation of the specific ribosome-binding activity of Lso2/CCDC124, indicate broadly important functions in translation and physiology.

## Author summary

Translation, or the production of protein from messenger RNA (mRNA), is catalyzed by a universally conserved macromolecular machine known as the ribosome. Ribosome-binding factors are also required for all substeps of translation, from initial recruitment of mRNA to peptide chain elongation to release of the mature polypeptide. However, many ribosome interactors have been identified whose effects on translation and physiology are unknown. Here, we show that the uncharacterized yeast protein late-annotated short open reading frame 2 (Lso2) crosslinks to a region of the ribosome that underlies accurate progression through all substeps of translation, the GTPase activation center. This specific binding activity is conserved in the human ortholog of Lso2, coiled-coil domain containing 124 (CCDC124). Null mutants of *lso2* also show severe translation defects during recovery from extended starvation, including failure to initiate on most mRNAs and a general block to peptide chain elongation. We propose that these defects could arise from a function for Lso2 in modulating the activity or integrity of the ribosome GTPase activation center during challenging growth regimes.

## Introduction

The ribosome is a universally conserved, multi-megadalton machine that carries out protein synthesis in all organisms. Accurate and efficient translation in eukaryotes requires the interaction of nearly 30 translation factors with the ribosome to ensure orderly execution of the translation cycle, including initiation, elongation, termination, and recycling of ribosomal subunits. Structural and biochemical studies have elucidated the interactions of these factors with the ribosome, and in many cases, their molecular functions are well understood. An intriguing and growing number of ribosome-associated proteins have been added to this core class, but their roles and mechanisms are generally less well characterized.

The known functions of ribosome-binding proteins underscore the broader connections of protein synthesis to homeostasis in all domains of life. Some examples include factors for quality control of defective mRNAs [1–3] and nascent peptides [4,5], regulators of gene-specific translation [6,7], signaling effectors of nutrient status at the ribosome [8,9], and modulators of ribosome activity in response to nutrient status [10–13]. Detailed functional characterization of these proteins has been illuminating in two ways. First, it has reinforced our understanding of the core translation cycle, for example, by demonstrating structural and mechanistic parallels between premature termination by quality control proteins and normal termination by release factors [14,15]. Second, ribosome-associated proteins have revealed new links between global adaptive responses and their specific effects on the ribosome. This point is particularly well illustrated in bacteria, in which stress-induced proteins affect each stage of the translation cycle by direct ribosome binding. For example, initiation is broadly inhibited by the ribosome hibernation factors YfiA (protein Y), hibernation promoting factor (HPF), and ribosome modulating factor (RMF), which sterically occlude translation ligands from their binding sites [12,13,16]. In addition, the status of elongation is monitored by RelA, which synthesizes the alarmone (p)ppGpp upon interaction with deacylated tRNA in the A site [8,9]. Activation of the stringent response by (p)ppGpp then reprograms metabolism and gene expression by causing large changes to the translatome, the available amino acid pools, and the fidelity of translation [17,18], which together promote adaptation to starvation. Finally, toxins that cleave mRNA in the A site of the ribosome elicit premature termination by quality control factors as a form of gene expression regulation during stress [19,20].

Unicellular eukaryotes such as *Saccharomyces cerevisiae* experience equally extreme shifts in nutrient status as do bacteria, and all cells need to adapt to acute stresses. Posttranslational modifications of core initiation factors and their regulators are the best-characterized mechanisms for widespread translational control (reviewed in [21]), but changing the composition or modification state of ribosomal complexes may also play a role [22,23]. Improvements in mass spectrometry have now expanded our ability to identify proteins in mature ribosomal complexes [24–27]. In mammalian cells, these include enzymes involved in ribosomal RNA (rRNA) modification, posttranslational protein modification, and carbon metabolism, with a potential moonlighting function for pyruvate kinase in translational control [26]. A proteomic analysis of mature ribosomes in rapidly dividing yeast also identified dozens of previously uncharacterized translation machinery–associated proteins [24], some of which are now known to play conserved roles in translation initiation and ribosome-associated quality control [5,28]. However, the compositions of eukaryotic ribosomal complexes in other growth states and cell types remain to be elucidated.

Here, we report the discovery and characterization of a novel and conserved ribosome-bound protein, late-annotated short open reading frame 2 (Lso2). By quantitative proteomics, Lso2 associates with yeast ribosomes in glucose-rich and glucose-starved conditions. We used transcriptome-wide crosslinking and immunoprecipitation (IP) to show that Lso2 interacts with tRNAs and with a specific region of the 25S rRNA near the A site. This inter-subunit binding site was validated by biochemical evidence that purified Lso2 promotes subunit association in vitro. Notably, Lso2's crosslink cluster in the 25S rRNA overlaps the universally conserved GTPase activation center (GAC) of the large subunit, a binding activity that we show is conserved in its human ortholog coiled-coil domain containing 124 (CCDC124). Lso2 is required for global translation during recovery from stationary phase, with *LSO2* nulls (*lso2Δ*) accumulating monosomes to abnormally high levels. Ribosome profiling in this condition revealed that both empty monosomes and translating 80S stalled at the start codon are increased in *lso2Δ*, with other elongation defects evident in gene bodies. These data suggest that Lso2 has a broadly required function in modulating the activity of eukaryotic ribosomes.

## Results

### Quantitative proteomics of ribosomes from two growth states identifies ribosome association of Lso2

**Fig 1:**
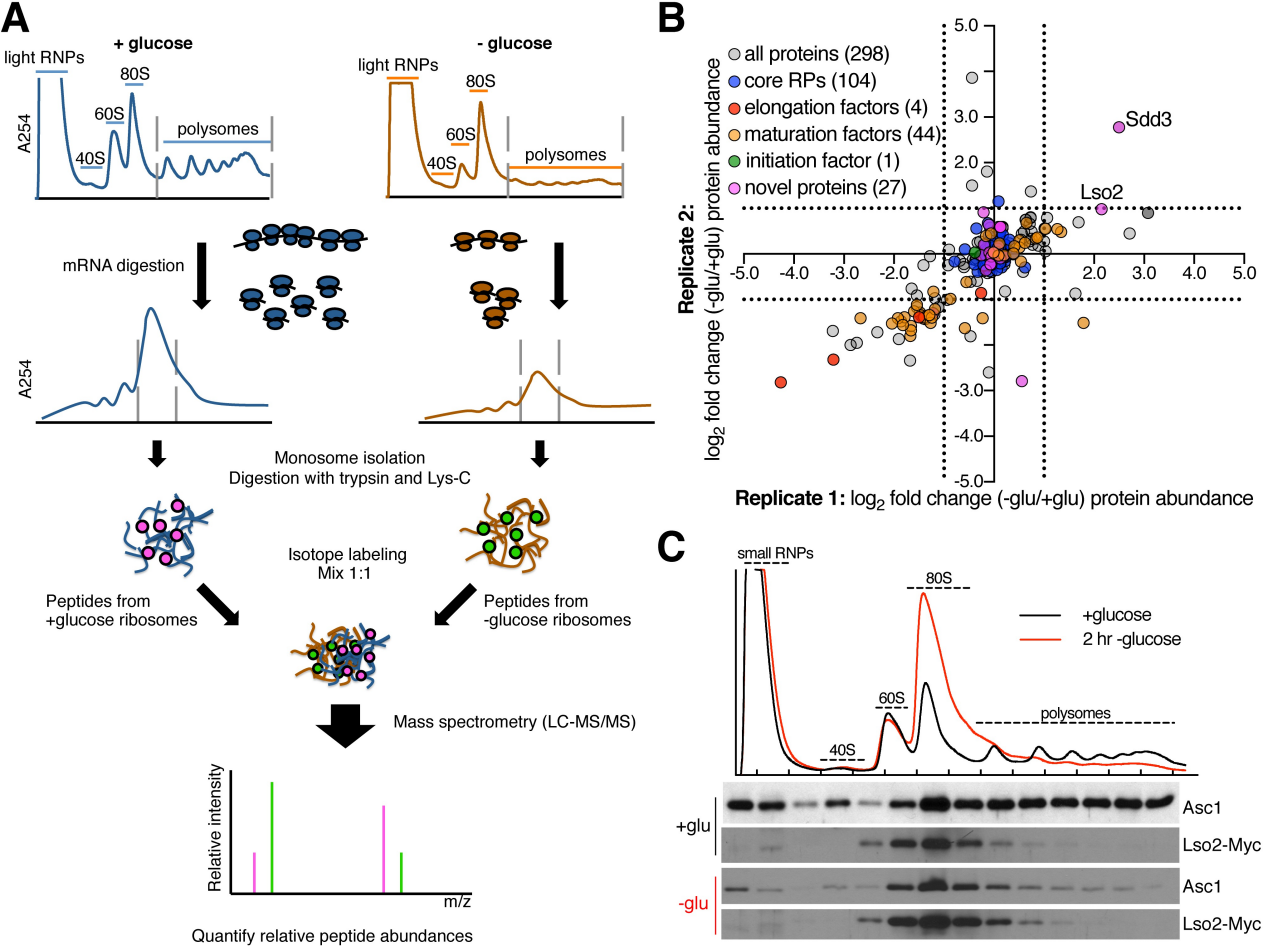
Lso2 associates with yeast ribosomes in two growth conditions. (A) Overview of method for purifying yeast ribosomes from glucose-replete and glucose-starved conditions for analysis by mass spectrometry. (B) Abundance changes in the ribo-proteome upon glucose starvation. Each axis, which represents 1 of 2 biological replicates, shows the log_2_ fold change in protein abundance at 2 hours of glucose starvation versus during log phase. The core RPs as a cohort are clustered at the origin, which enables quantification of changes in the stoichiometry of other proteins relative to the ribosome. The dashed lines demarcate abundance changes greater than 2-fold. (C) A Myc-tagged *LSO2* strain was grown to log phase in YPAD (+glucose) or to log phase in YPAD and then shifted to YPA (lacking glucose) for 2 hours (2 hr -glucose). Cells were harvested for fractionation through a sucrose gradient, and fractions were probed for the Myc epitope and for the core 40S protein Asc1 as a loading control. See also S1 Fig and S1 Table. LC-MS/MS, liquid chromatography–tandem mass spectrometry; Lso2, late-annotated short open reading frame 2; RNP, ribonucleoprotein; RP, ribosomal protein; Sdd3, suppressor of degenerative death 3; YPA, yeast extract, peptone, adenine medium; YPAD, YPA with glucose.

Glucose withdrawal alters the transcription, translation, and translational efficiencies of the majority of genes in budding yeast [29–31]. However, despite the magnitude of the translational response to glucose withdrawal, these translation changes do not depend on the canonical cap-binding or general control pathways that target functional levels of initiation factors [29]. We therefore hypothesized that the composition of the ribosome itself might be altered. To test this, polyribosomes were isolated from glucose-starved and replete conditions and compared by quantitative mass spectrometry (Fig 1A, Materials and methods). To increase the purity of ribosome complexes, polysomal fractions were digested with limited RNase and fractionated on a second gradient to obtain 80S monoribosomes. Peptide digests of equal mass of purified monosomes from glucose-starved and replete conditions were then differentially labeled with nonisobaric mTRAQ [32] amine-labeling reagents prior to analysis by tandem mass spectrometry (MS/MS).

Two hundred ninety-eight proteins met the requirements for quantitative comparison between samples, based on identification by ≥2 unique peptides and quantification of ≥2 peptide ratios. Protein abundance changes were reproducible overall between independent biological replicates (R^2^ = 0.49; Fig 1B; S1 Table). Seventy-eight of 79 core ribosomal proteins (RPs) were quantified, including 22 of 38 RP paralogs that differ by at least 1 amino acid. As expected based on 1:1 mixing of total protein from each condition, RPs were equally abundant in starved and replete samples. Initiation factors were mostly absent, which is consistent with their unstable association with ribosomes in the absence of crosslinking [33]. Eukaryotic elongation factor 1A (eEF1A [*TEF2*]), eukaryotic elongation factor 1Bγ (eEF1Bγ [*TEF4*]), eukaryotic elongation factor 2 (eEF2 [*EFT2*]), and eukaryotic elongation factor 3 (eEF3 [*YEF3*]) were recovered at lower levels from starved polysomes. Forty-four ribosome biogenesis factors, which are known to associate with complexes larger than 80S [34,35], were also quantified. These generally showed reduced abundance in starved samples, consistent with decreased ribosome biogenesis under these conditions [31]. Other proteins that were reduced in glucose-starved samples include glycolytic enzymes that are abundant in replete conditions. These are likely contaminants, although we cannot exclude a moonlighting function for these proteins in translation [36]. Thus, our gradient purification and mass spectrometry achieved high coverage of core RPs and recovered known translation factors within ribosomal complexes.

We also identified several previously uncharacterized proteins as novel ribosome-binding factors. Notably, Lso2 and suppressor of degenerative death 3 (Sdd3) reproducibly showed greater relative enrichment in starved polysome samples (Fig 1B, S1 Table). Lso2 is a small basic protein predicted to contain a coiled-coil domain (92 amino acids, 10.5 kDa, pI = 10.78) (S1A Fig) [37,38]. Polysome western blots confirmed the comigration of Lso2 with ribosomes in both rich medium and glucose starvation (Fig 1C). Strikingly, the entire cellular population of Lso2 is ribosome associated and enriched in the monosome fractions. In starved cells, the abundance of Lso2 in small polysomes was increased relative to the core 40S protein Asc1, which is consistent with our mass spectrometry results (Fig 1B and 1C). However, there was no change in Lso2 abundance relative to total protein during glucose starvation (S1B Fig). Addition of EDTA to dissociate polysomes and 80S monosomes caused Lso2 to shift to the free pool and subunit fractions, consistent with the behavior of a ribosome-associated protein (S1C Fig). Lso2 is expressed at one-tenth the level of core RPs in rapidly dividing cells, based on our ribosome profiling data [30], which is comparable to the estimated abundance of Lso2 from mass spectrometry–based proteomics [39]. Thus, it is plausible that most monosomes, which account for <15% of all ribosomes in growing yeast, contain Lso2 in glucose-grown cultures.

In contrast to Lso2, only a small minority of cellular Sdd3 comigrated with ribosomes in rich medium or starvation (S1D Fig). We subsequently focused on characterizing Lso2. This protein is conserved, with 63 predicted orthologs in 57 higher eukaryotes, including humans (S1A Fig) [40]. In budding yeast, the paralog Lso1 (Yjr005c-a) shares 79% similarity with Lso2 but is induced 1.5- to 20-fold by different iron starvation treatments [41,42], in contrast to the constitutively expressed Lso2 [41]. Consistently, the *LSO1* but not *LSO2* promoter contains binding sites for the iron-responsive activating transcription factors 1/2 (Atf1/2) [41,42]. Semiquantitative plate growth showed that *lso2Δlso1Δ* nulls are synthetically sick during iron starvation, suggesting their functions could overlap [41]. However, in replete growth, Lso1 is one-tenth the abundance of Lso2, as determined by western blotting of S288C [41], the yeast strain used in these previous studies [41,42]. This is corroborated by the ribosome profiling of [43], in which *LSO1* had 13.8 reads per kilobase per million reads (RPKMs) of expression, while *LSO2* had 299.4 RPKMs. Replete expression of *LSO1* is even lower in the Sigma1278b background used in this report. We found by western blot that Lso1 is undetectable in replete Sigma1278b, even in severely overexposed blots, and up-regulated by a functional inducer of iron starvation as expected (S1E Fig) [42]. Similarly, ribosome profiling of replete, wild-type (WT) Sigma1278b captured approximately 250 RPKMs for *LSO2* but 0.6 for *LSO1* [30]. While we cannot rule out that Lso1 is expressed beneath this detection threshold, we conclude that Lso2 is the dominant paralog under standard growth conditions. Thus, Lso2 is a novel ribosome-associated protein and likely to have a nonredundant, broadly required function in translation.

### Lso2 crosslinks to 25S rRNA near the A site and to tRNAs

**Fig 2:**
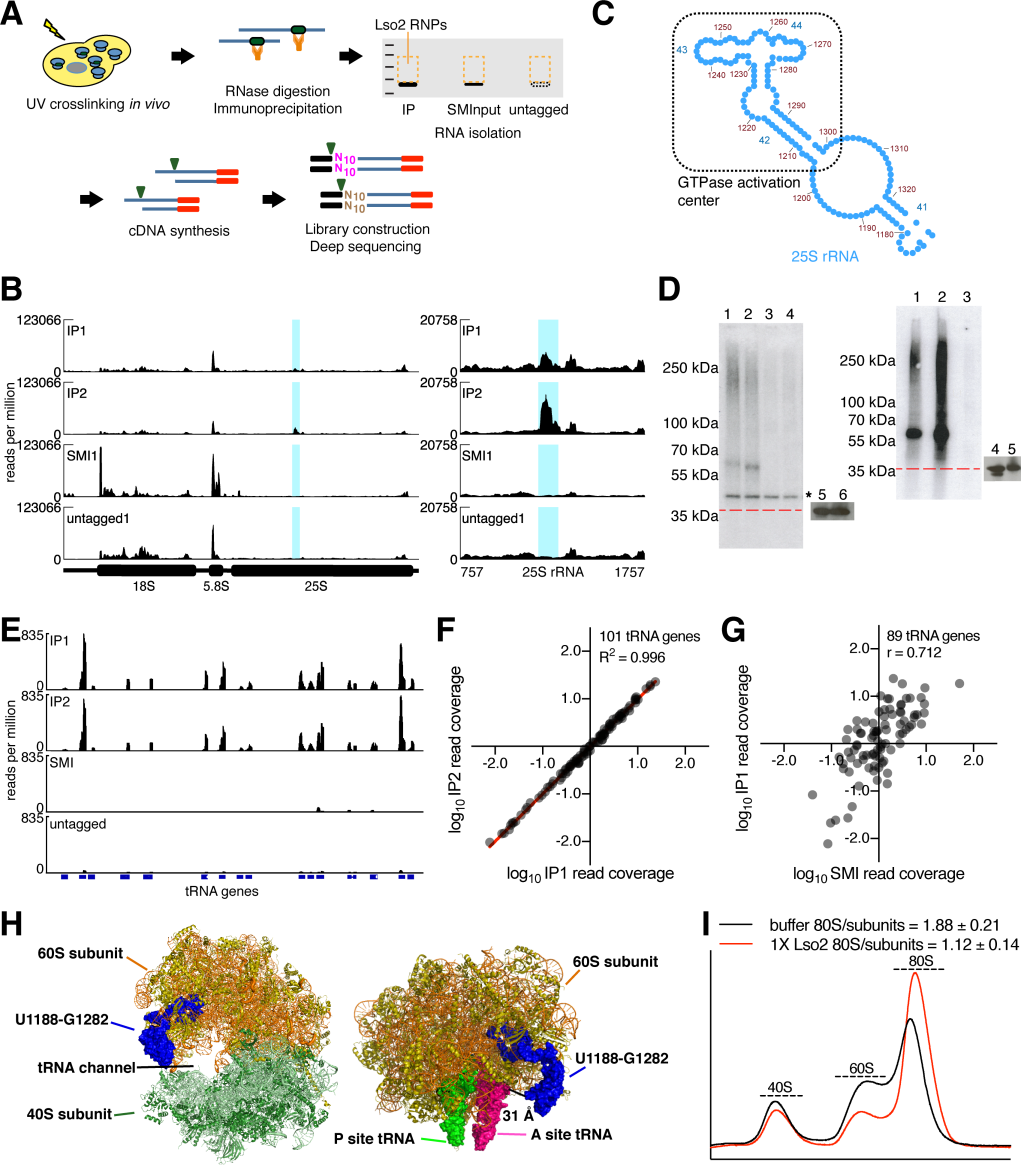
Lso2 crosslinks to 25S rRNA near the A site and to tRNAs. (A) Overview of ePAR-CLIP library construction. (B) (Left) Normalized read coverage across the *RDN37* locus in ePAR-CLIP libraries prepared with 1:20,000,000 RNase I. The y-axis is reads per million reads in the entire library. Two untagged IP libraries and 2 SMI libraries, each strain-matched to an IP replicate, were the controls in this set. One SMI and 1 untagged library are shown for clarity. Blue bars indicate the region that was significantly enriched (fold-enrichment > 4, *p* < 10^−5^) in both IP replicates relative to its paired SMI, as well as to the 2 untagged libraries. (Right) Inset of normalized read coverage across the enriched region in *RDN25*. (C) Secondary structure of the subregion of 25S rRNA [47] containing the Lso2 crosslink cluster. Helices 42, 43, and 44 comprise the GTPase activation center. (D) (Left) Diagnostic electrophoresis membrane of radiolabeled Lso2-RNPs from ePAR-CLIP libraries prepared with 1:20,000,000 RNase I. The red line indicates the position of Lso2-Myc alone (without crosslinked RNAs), based on the positions of protein markers. Lanes 1 and 2, IP replicates; lanes 3 and 4, untagged replicates; lanes 5 and 6, western blots of Lso2-Myc in 0.5% of the input and in 13% of the IP, respectively. The region from 50 kDa to approximately 130 kDa was excised for each sample. Asterisk indicates a nonspecific labeled RNA species present in all libraries. (Right) As in left, except from ePAR-CLIP libraries prepared with 1:2,000,000 RNase I. Lanes 1 and 2, IP replicates; lane 3, untagged; lanes 4 and 5, western blots of Lso2-Myc in 0.5% of the input and in 13% of the IP, respectively. The region from 35 kDa to 100 kDa was excised for each sample. (E) Normalized read coverage across a series of negative strand tRNA features in ePAR-CLIP libraries prepared with 1:2,000,000 RNase I. The y-axis is reads per million reads in the entire library. Blocks represent exons, while thin lines represent introns. (F) Correlation of tRNA read densities between the IP replicates made with 1:2,000,000 RNase I. Read density values were median centered and log_10_ transformed. R^2^ of linear fit is indicated. (G) Correlation of tRNA read densities between IP replicate 1 versus the SMI for ePAR-CLIP libraries made with 1:2,000,000 RNase I. Read density values were median centered and log_10_ transformed. (H) (Left) The rRNA crosslink cluster from (B) on the crystal structure of the 80S ribosome ([48], PDB 4V88). (Right) The identical cluster on a cryo-EM structure of the 60S subunit containing P and A site tRNAs in the classic state ([49], PDB 5GAK). (I) Lso2 stabilizes ribosomal subunit association in vitro. One μM each (100 pmol) of 40S subunits and 60S subunits purified from yeast *lso2Δ* was mixed with 1 μM (100 pmol) of purified recombinant Lso2 or with an equivalent volume of buffer. The mixture was incubated at 37°C for 10 minutes before fractionation through a sucrose gradient. The ratio of 80S ribosomes to the sum of 40S and 60S subunits was quantified. *n* = 3 technical replicates; mean ± S.D. See also S2 Fig and S2 Table. EM, electron microscopy; ePAR-CLIP, photoactivatable ribonucleoside crosslinking and immunoprecipitation and an enhanced method of CLIP library preparation; IP, immunoprecipitation; Lso2, late-annotated short open reading frame 2; Lso2-Myc, yeast strains expressing Myc-tagged Lso2 at endogenous levels; PDB, Protein Data Bank; RNP, ribonucleoprotein; rRNA, ribosomal RNA; SMI, size-matched input.

We next sought to validate Lso2's ribosome association and to identify its ribosomal binding site with higher resolution. We used photoactivatable ribonucleoside crosslinking and IP and an enhanced method of CLIP library preparation (ePAR-CLIP) to identify RNAs genome-wide that crosslink to Lso2 in vivo (Fig 2A, Materials and methods) [36,44]. Briefly, yeast strains expressing Myc-tagged Lso2 at endogenous levels (Lso2-Myc) were grown with 4-thiouracil and irradiated at 365 nm. We verified that these manipulations do not affect Lso2's pattern of ribosome co-sedimentation (S2A Fig). Cells were then lysed and digested with limited RNase I, followed by IP of Lso2-Myc and crosslinked RNA. Lso2-ribonucleoprotein (RNP) complexes from 2 independent biological replicates were further purified by SDS-PAGE in parallel with a size-matched input (SMI), which was processed identically except for omission of the IP step, and with an anti-Myc IP of a strain lacking the Myc epitope (hereafter called the untagged). Previous work has shown that comparison to SMI and untagged negative controls eliminates the majority of CLIP peaks as false positives [44,45]. Following deep sequencing, reads were collapsed to remove PCR duplicates and mapped to a modified version of the yeast genome containing single copies of the rDNA locus and each unique tRNA gene. We used a previously published peak identification algorithm [46] and stringent criteria to identify clusters reproducibly enriched in the IP versus both control libraries (Materials and methods).

A single 95-nucleotide cluster in the 25S rRNA, U1188 to G1282, was the only region of the transcriptome to be reproducibly enriched >4-fold (*p* < 10^−5^) (Fig 2B). We also observed a peak of T-to-C mutation frequency at U1253, which was reproducible in both IPs and absent from the controls (S2B Fig). These transitions reflect bona fide crosslinking of 4-thiouridine leading to preferential misincorporation of dG during reverse transcription [50]. Our mutation analysis also demonstrates the utility of SMI and untagged controls, which allowed identification of crosslink residues that are recovered nonspecifically (S2B Fig). The region U1188 to G1282 overlaps the GAC, composed of helices 42, 43, and 44 of the 25S rRNA (Fig 2C). In an effort to further narrow the crosslink cluster, a second set of libraries was prepared using 10-fold more RNase. However, under these conditions, no subregions of rRNA met the enrichment criteria, suggesting that increased RNase digestion depleted larger and more labile rRNA fragments from the IP samples. Instead, about 90% of all reads in each of these Lso2-Myc IP replicates mapped to tRNA genes (Fig 2E and S2C Fig). Consistent with this read distribution, the prominent band (approximately 60 kDa) visible in ^32^P-labeled Lso2-Myc RNP IPs was of the expected size for a complex containing Lso2-Myc bound to an intact tRNA (Fig 2D, right). We verified that RNPs crosslinked to a canonical tRNA-modifying enzyme, pseudouridine synthase 1 (Pus1), show a similar upshift of about 25 kDa compared to the protein alone (S2D Fig). Together with the fact that Lso2 is undetectable in subribosomal gradient fractions (Fig 1C and S2A Fig), these results suggest that Lso2 associates with tRNA-bound ribosome complexes in vivo.

Given that the length of an entire tRNA gene is the size of subfeatures typically identified as peaks [46], we looked for tRNAs with ≥4-fold enrichment of read density in both IPs versus the SMI and untagged. Eighty of 101 total tRNA genes met the minimum read cutoff (64 reads in all 4 libraries) for this analysis. Of these, 70 were enriched ≥4-fold (S2 Table). The read densities of tRNAs were well correlated between IP replicates (R^2^ = 0.996, Fig 2F), and the relative abundances in each IP were positively correlated with those in the SMI (Pearson r > 0.7, Fig 2G and S2E Fig), indicating a broad capacity for Lso2 to interact with tRNAs on the ribosome. The 25S binding site of Lso2 is within 30 Å of a classical A site tRNA (Fig 2H) [49], which easily positions a protein the size of Lso2 to interact with A site tRNAs [51]. Depending on its shape or multimerization status, Lso2 could also reach to the P site tRNA of a ribosome with an empty A site. Taken together, these data suggest that Lso2 is able to associate with tRNA-bound ribosomes by binding in the tRNA channel on the A site side of the 60S subunit.

To validate the ribosomal binding site of Lso2, we tested whether it is able to stabilize ribosomal subunit association in vitro. This biochemical function was previously demonstrated for the bacterial ribosome hibernation factors, a class of ribosome-binding proteins with sizes similar to Lso2 and which likewise bind directly in the tRNA channel. For the gradient association assay [52], individual ribosomal subunits were isolated from *lso2Δ*, and recombinant His-tagged Lso2 (6XHis-Lso2) was purified from *E*. *coli* (S2F Fig). 40S and 60S subunits were mixed in an equimolar ratio, to which equimolar Lso2 or the equivalent volume of buffer was added before fractionation on sucrose gradients to quantify the distributions of monosomes versus free subunits (Materials and methods). At near-physiological magnesium concentrations (3 mM), we observed a characteristic distribution of free ribosomal subunits and 80S monosomes. Inclusion of equimolar Lso2 reproducibly increased monosome formation by 2-fold (Fig 2I). These data validate direct ribosome binding by Lso2 and are consistent with binding near the tRNA channel, as shown by ePAR-CLIP.

### The specific ribosome-binding activity of Lso2 is conserved to humans

**Fig 3:**
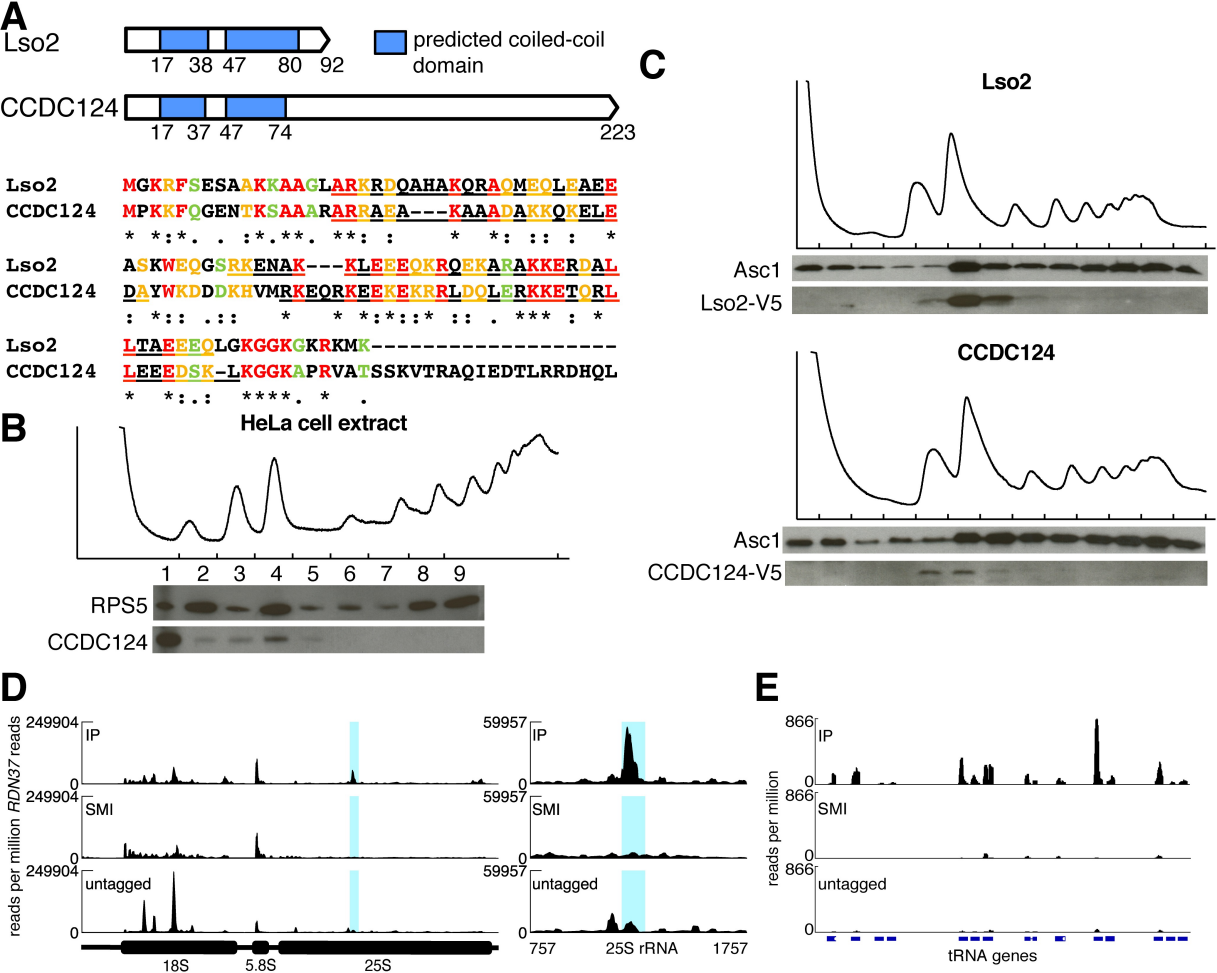
The ribosome-binding activity of yeast Lso2 is conserved in its human ortholog. (A) (Top) Schematic of coiled-coil domains in Lso2 and CCDC124. (Bottom) Protein sequence alignment of yeast Lso2 with human CCDC124. (B) HeLa cell extract was fractionated through a sucrose gradient containing 5 mM magnesium. Equivalent fraction volumes were TCA precipitated and loaded in each lane. Fractions were probed for RPS5 and for endogenous CCDC124. (C) For both strains, cell extracts were fractionated through a sucrose gradient and the fractions probed for Asc1 and V5. (Top) The yeast *LSO2* gene was tagged with V5 in a marker-free insertion. (Bottom) The yeast *LSO2* gene was swapped with V5-tagged *CCDC124* in a marker-free replacement. (D) (Left) Internally normalized read coverage across the *RDN37* locus in CCDC124-Myc ePAR-CLIP libraries. The y-axis is reads per million reads mapping to *RDN37*. The blue region is the 25S rRNA cluster that reproducibly crosslinked to Lso2-Myc (Fig 2B). (Right) Inset of *RDN37-*normalized read coverage across the *RDN25* region crosslinking to Lso2-Myc. (E) Normalized read coverage across a series of negative-strand tRNA features in CCDC124-Myc ePAR-CLIP libraries. Y-axis is reads per million reads in the entire library. See also S3 Fig and S3 Table. CCDC124, coiled-coil domain containing 124; ePAR-CLIP, photoactivatable ribonucleoside crosslinking and immunoprecipitation and an enhanced method of CLIP library preparation; IP, immunoprecipitation; Lso2, late-annotated short open reading frame 2; Lso2-Myc, yeast strains expression Myc-tagged Lso2 at endogenous levels; RPS5, ribosomal protein S5; TCA, trichloroacetic acid.

We identified putative orthologs of yeast Lso2 in multiple eukaryotes, suggesting a conserved function. The human ortholog of Lso2, CCDC124, is a poorly characterized protein suggested to localize to the midbody during cytokinesis in HeLa cells [53] and to interact with Protein Kinase R during innate immune stimulation of HEK293 cells [54]. CCDC124 was independently predicted to contain a coiled-coil domain at its N-terminus (Fig 3A) [37,38] and is ubiquitously expressed across two dozen human tissues profiled by proteomics [55]. Additionally, CCDC124 copurified with crosslinked poly(A) RNA from human Huh7 and HeLa cells [36], suggesting an association with cytoplasmic mRNPs. Although CCDC124 has not been characterized as ribosome-associated, an interactome survey of 1,125 mammalian proteins tagged with green fluorescent protein (GFP) and expressed in HeLa cells found interactions between CCDC124 and two 60S RPs, RPL10 (human) and Rpl35 (mouse) [56].

To determine whether the putative human ortholog of Lso2 also associates with ribosomes in human cells, HeLa extracts were fractionated on a sucrose gradient and probed for endogenous CCDC124. While the majority of the protein sediments in the free (nonribosomal) pool, a minority is enriched in the monosome fraction (Fig 3B). This 80S subpopulation shifts entirely to the subribosomal, 40S, and 60S fractions in the presence of EDTA, as expected for a ribosome-associated protein (S3A Fig). Given that CCDC124, like *LSO2*, is expressed at levels substoichiometric to core ribosomes (HeLa ribosome profiling data of [57]), total abundance alone fails to explain this difference in its distribution compared to yeast Lso2. We conjecture that the human protein, which is 131 amino acids longer than Lso2 (Fig 3A), is regulated in a more complex manner vis-à-vis its extraribosomal versus monosome-bound subpopulations. Alternatively, CCDC124 may have additional and independent roles off the ribosome. These observations demonstrate that a subset of CCDC124 is ribosome-bound in human cells and suggest a conserved function in translation.

As additional evidence that the ribosome-binding activity of Lso2 is conserved, CCDC124 expressed in yeast associated with ribosomes in a manner similar to native Lso2. When yeast chromosomal *LSO2* was replaced with V5-tagged *CCDC124*, the human protein sedimented exclusively with 60S and 80S ribosomes (Fig 3C). A putative shorter isoform of CCDC124 [58] that contains 31 amino acids beyond the coiled-coil domain similarly comigrates with yeast ribosomes (S3B Fig). To identify the human CCDC124 binding site on yeast ribosomes, we performed ePAR-CLIP on Myc-tagged CCDC124 expressed from the yeast *LSO2* locus (S3C Fig). We observed a peak of internal enrichment at 25S U1188 to G1282 (Fig 3D), which is where Lso2 reproducibly crosslinked to rRNA (Fig 2B), and an IP-specific peak of T-to-C mutation frequency at 25S U1253 (S3D Fig), as was observed with Lso2. CCDC124 also crosslinked to a broad range of tRNAs (Fig 3E): of 80/101 tRNA genes meeting the read cutoff, 66 were enriched in the CCDC124 IP relative to both controls (S3 Table). Of these, 65 are also targets of Lso2 (S2 Table). These results suggest that CCDC124 and Lso2 share a specific ribosome-binding activity.

### Translation is globally depressed in *lso2Δ* recovering from starvation

**Fig 4:**
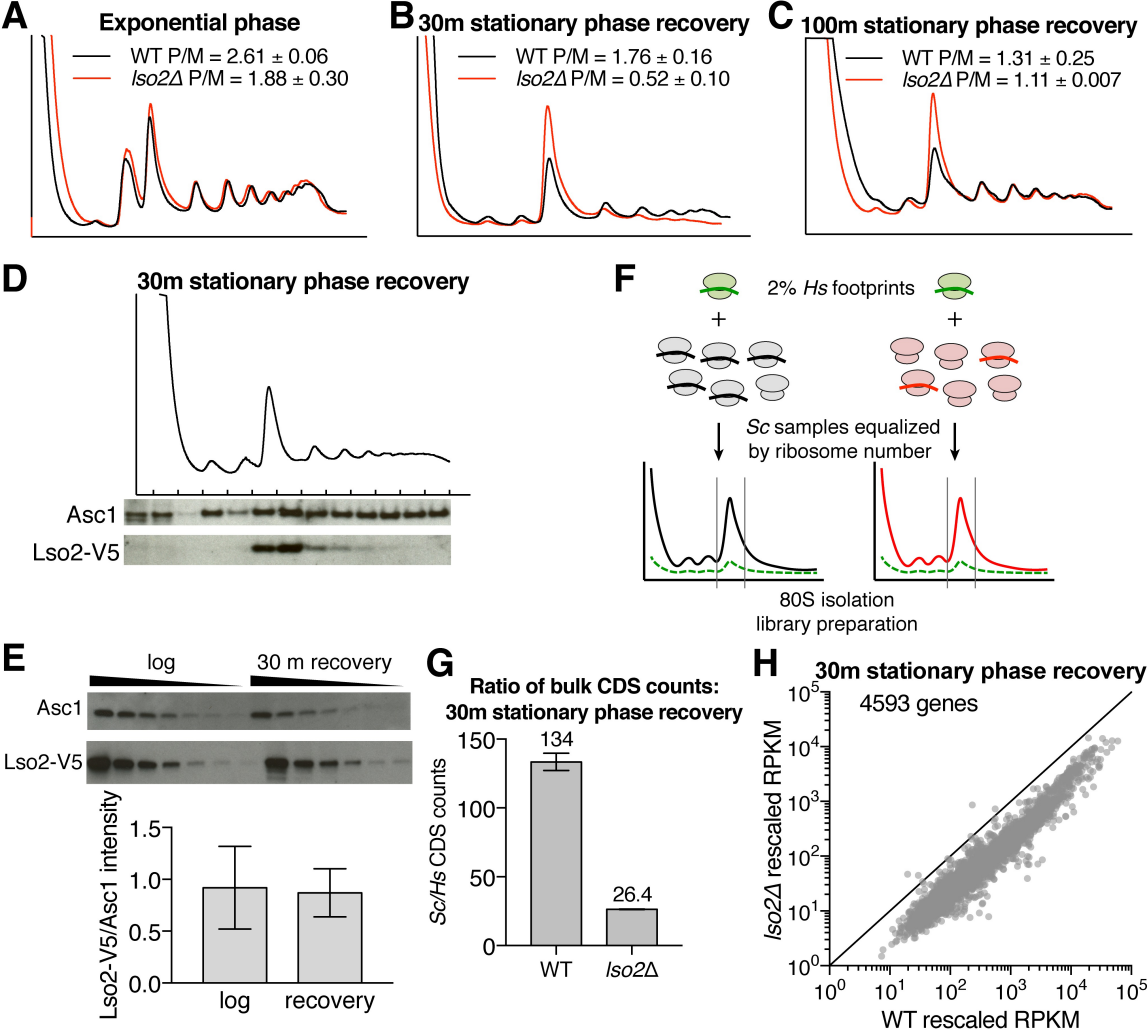
Lso2 is required for global translation during recovery from stationary phase. (A) Gradient profiling of WT and *lso2*-null strains during exponential phase, with quantification of the polysome-to-monosome ratios (“P/M”). *n* = 3 biological replicates; mean ± S.D. (B) WT and *lso2Δ* were grown in YPAD for 4 days and then transferred to fresh YPAD for 30 minutes before harvesting for gradient profiling. The polysome-to-monosome ratios of each strain are quantified. *n* ≥ 2 biological replicates; mean ± S.D. (C) As in (B), except that WT and *lso2Δ* were recovered for 100 minutes in YPAD following stationary phase. *n* = 2 biological replicates; mean ± S.D. (D) V5-tagged *LSO2* strains were grown in YPAD for 4 days and then transferred to fresh YPAD for 30 minutes before harvesting for gradient profiling. Gradient fractions were probed for the V5 epitope and for Asc1. Shown are representative data from 2 biological replicates. (E) V5-tagged *LSO2* strains were grown either to log phase or for 4 days followed by 30 minutes of recovery in fresh YPAD. Extracts from each sample were loaded in a 2-fold dilution series and probed for the V5 epitope and for Asc1. The slope of signal intensity versus dilution factor was used as the abundance of each protein (Materials and methods). *n* ≥ 1 technical replicate for each of 2 biological replicates; mean ± S.D. (F) Overview of ribosome profiling method using internal standards for absolute comparisons of mRNA abundances between conditions. (G) The ratio of all unique yeast to all unique human CDS-mapping read counts in each condition, which is a measure of global protein synthesis. *n* = 2 biological replicates; mean ± S.D. (H) Yeast RPKMs from each library were rescaled using the slope of the linear regression of human RPKMs from that library versus the normalizing library (fixed as *lso2Δ* replicate 1). Plotted are the averages of rescaled RPKMs from the 2 *lso2Δ* replicates versus the averages of rescaled RPKMs from the 2 WT replicates. See also S4 Fig. CDS, coding sequence; Lso2, late-annotated short open reading frame 2; RPKM, read per kilobase per million reads; WT, wild type; YPAD, yeast extract, peptone, adenine medium with glucose.

The binding site of Lso2 near the GAC suggests its potential to affect multiple stages of the translation cycle. We therefore tested whether loss of Lso2 perturbs overall growth or global translation in various conditions. Bulk polysomes were normal in *lso2Δ* under replete conditions and after 3 hours of glucose starvation (Fig 4A and S4A Fig). Growth was also normal at various temperatures and on different carbon sources (S4B Fig). Next, we examined *lso2Δ* during translational recovery from stationary phase, a challenging condition in which a reduced number of cellular ribosomes must rapidly resynthesize the growth-promoting proteome [59,60]. WT and *lso2Δ* strains were grown to nutrient exhaustion by culturing without dilution for 4 days and then transferred to fresh medium for 30 minutes before gradient profiling. *lso2Δ* showed a 3-fold accumulation of monosomes at the expense of polysomes (Fig 4B), with polysome levels partially recovered after 100 minutes of upshift (Fig 4C). Both WT and *lso2Δ* required 5 hours before the first cell division (S4C Fig), which is consistent with *lso2Δ*’s attenuated defect by 100 minutes. To determine where Lso2 sediments during recovery, we probed for V5-tagged Lso2 in sucrose gradient fractions after 30 minutes of upshift. The enrichment of Lso2 in 80S fractions was similar to that in replete conditions (compare Figs 1C and 4D), although Lso2 is required for bulk translation only during recovery. The stoichiometry of Lso2 to core ribosomes also remained similar to that of log phase (1:10, Fig 4E).

The 80S fraction can include nontranslating monosomes, translating monosomes engaged on mRNA, and monosomes generated by endonucleolytic cleavage of mRNA during no-go and non-stop decay [3,61–63]. To distinguish between these species and to examine other translation defects in recovering *lso2Δ*, we performed ribosome footprint profiling [64]. At 30 minutes of stationary phase upshift, yeast cells were harvested by rapid filtration and freezing without pretreatment with cycloheximide (Materials and methods) [65], which is known to distort specific codon pauses and lead to artefactual accumulation of ribosomes at start codons [66,67]. RNase-digested HeLa lysate was added in fixed proportion to the number of yeast ribosomes in each sample as an internal standard (Fig 4F, Materials and methods) [68]. Subsequent normalization of yeast to human coding sequence (CDS) counts thereby enabled absolute comparisons of mRNA abundances between samples. To improve quantification, a randomized barcode was introduced at the 5′ end of each read for PCR duplicate collapsing. Two independent biological replicates of both WT and *lso2Δ* were profiled with reproducible results (Pearson r^2^ > 0.97, S4D Fig), yielding translation data for 4,593 yeast genes that had sufficient reads across all libraries.

Normalizing the bulk count of yeast to human CDS-mapping reads revealed a 5-fold decrease in overall translation in *lso2Δ* relative to WT (Fig 4G). To determine how this reduction affected genes individually, we applied a global scaling factor to the yeast RPKM values in each library, which was calculated as the slope of the linear regression of human RPKMs from that library versus the normalizing library (fixed as *lso2Δ* replicate 1, S4E Fig) [69]. Overall, the absolute number of translating ribosomes was substantially reduced for most genes (Fig 4H). Given that the total number of cellular ribosomes was unchanged (S4G and S4H Fig), this global reduction in translating ribosomes shows that the increased 80S peak in *lso2Δ* is comprised mainly of nontranslating monosomes. This interpretation is consistent with the fact that most of the 80S peak dissociates in high salt (S4F Fig), which preferentially disrupts monosomes devoid of mRNA and translation ligands [70]. Because ribosomal subunits must complete the multistep initiation pathway to be stably engaged on mRNA, the abnormally high fraction of empty subunits suggests a bottleneck to one or more steps of initiation in *lso2Δ*. Together, these results reveal a critical role for Lso2 during translational recovery.

Given the global depression of translation observed in recovering *lso2Δ*, we considered the possibility that increased expression of Lso1 might compensate for loss of Lso2 under other conditions in which there was no phenotype. We therefore examined *LSO1* expression in *lso2Δ* under replete conditions, after 3 hours of glucose starvation, and during recovery from stationary phase. Each of these regimes showed ≤1 read count from the paralog *LSO1* despite coverage of >82% of the transcriptome. While we cannot rule out that some Lso1 may be expressed beneath our detection limit, Lso1 is unlikely to be at sufficient levels to compensate for the absence of *LSO2*. For comparison, in the matched WT libraries from these conditions, *LSO2* was above the ≥65th percentile of expression based on RPKM normalization of ≥422 raw reads, whereas ≤1 read mapped to *LSO1*. Thus, Lso2 is specifically required for normal cellular translation during recovery from stationary phase.

### Stored ribosomes are defective for translational recovery in *lso2Δ*

A previous high-throughput screen of synthetic genetic interactions in yeast identified several ribosome biogenesis factors as among the strongest negative interactors with *lso2Δ* [71]. We therefore considered whether the observed translation defects in *lso2Δ* might be downstream of a failure to make new and functional ribosomes by 30 minutes of upshift. According to this model, the polysomes in WT cells would consist primarily of newly synthesized ribosomes, whereas newly synthesized ribosomes in *lso2Δ* fail to enter the translation cycle. Quantification of rRNA per cell in WT and *lso2Δ* at 0 and 30 minutes, however, showed that neither strain increased its mature rRNA content during this time (S4G Fig). Thus, stored ribosomes carry out the observed translation program, which indicates that the translation defects in *lso2Δ* are likely not downstream of a failure to produce functional new ribosomes.

We also examined the bulk integrity and quantity of rRNA stored in *lso2Δ* after 4 days of stationary phase, which appeared normal (S4H Fig). However, we cannot rule out subtler defects in the stored ribosomes. Indeed, after stationary phase, 60S subunits from *lso2Δ* sedimented aberrantly following the RNase I digestion step of ribosome profiling (S4I Fig), which is consistent with the possibility that lack of Lso2 affects the proper storage of ribosomes. If so, the mechanism is likely to be indirect, since Lso2 remains substoichiometric to ribosomes during stationary phase. The observed genetic interactions between *lso2Δ* and biogenesis factors [71] may reflect synthetic effects of reducing ribosome number and compromising their function (Discussion).

### Absence of Lso2 perturbs early elongation

**Fig 5:**
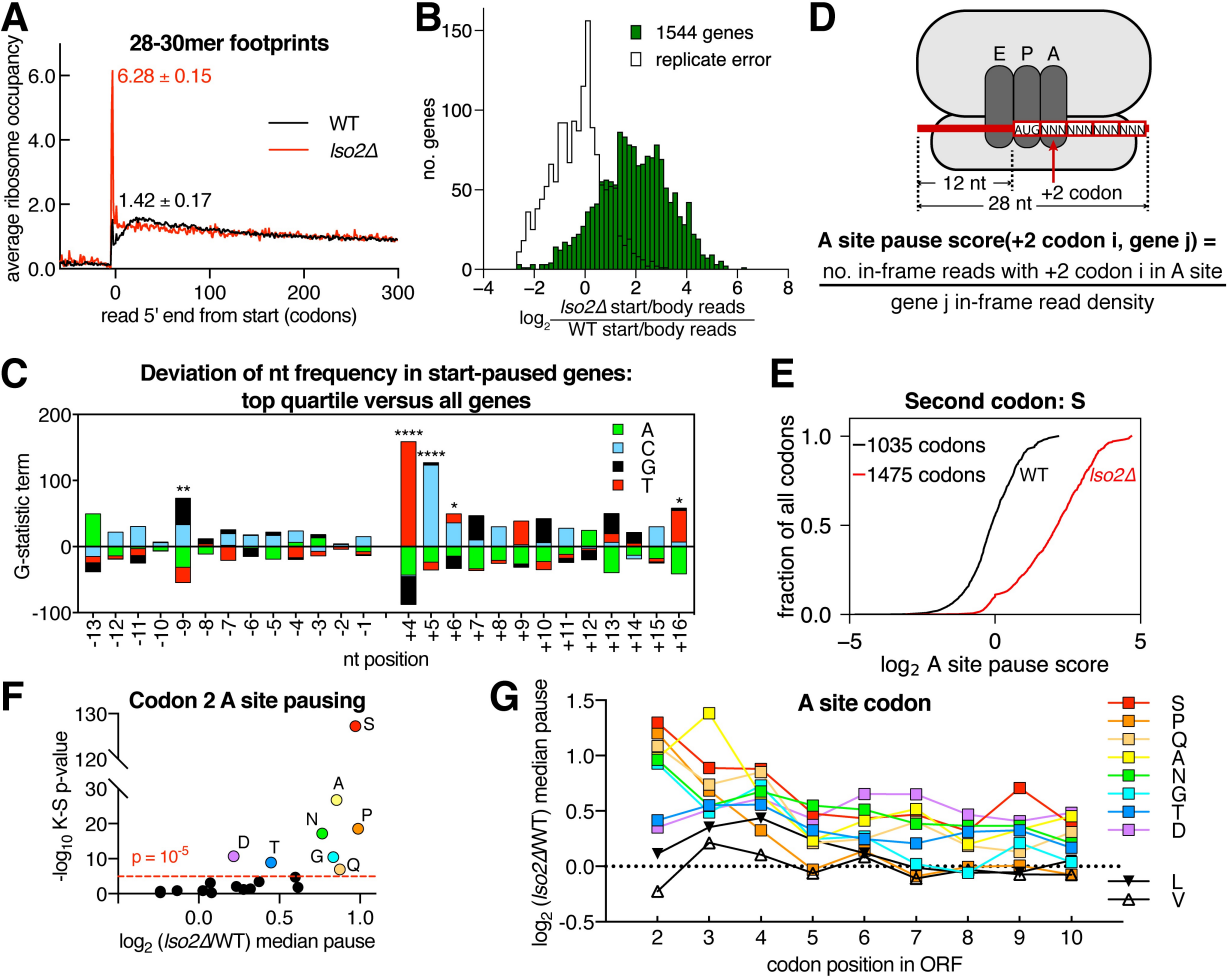
Absence of Lso2 perturbs early elongation. (A) Metaribosome occupancy in a window around the start codon for 28–30 mer footprints. Numbers indicate the respective start codon occupancy in WT and *lso2Δ*. *n* = 2 biological replicates; mean ± S.D. (B) Histogram of change in the ratio of start codon to ORF body footprints in *lso2Δ* versus WT for each gene with ≥64 reads. Black trace (replicate error) indicates the same comparison for WT replicate 1 versus replicate 2. (C) Nucleotide usage bias in the top quartile of genes shown in (B). −1 and +4 indicate the first nucleotide upstream and downstream of the start codon, respectively. At each position, the G-statistic contribution of nucleotide *N* was calculated as $2O^{N}ln(\frac{O^{N}}{E^{N}})$, where *O*^*N*^ is the observed counts of *N* usage in the top quartile, and *E*^*N*^ is the expected counts based on the usage frequency of all genes analyzed. Bonferroni-corrected *p*-values are indicated by asterisks. *, *p* < 0.05; **, *p* < 0.01; ***, *p* < 0.001, ****, *p* < 0.0001. (D) Anatomy of ribosome footprint used to calculate pause scores of codon 2 in the A site. The number of read 5′ ends was divided by the density of all in-frame reads within the encoding gene. (E) Empirical cumulative distribution of pausing at +2 serine codons in the A site, which are challenging to translate in *lso2Δ*. Each trace is from merged WT and *lso2Δ* replicates, respectively. (F) For each indicated +2 codon in the A site, empirical cumulative distributions of pause scores were computed for WT and *lso2Δ* as in (E). The K-S *p*-value and median shift of *lso2Δ* versus WT pause-score distributions are plotted. The 8 most challenging +2 codons (median increase > 1.78-fold, *p*-value < 10^−6^) are highlighted. (G) For the 8 challenging +2 codons highlighted in (F), the median pause shift was also calculated in the same manner for each of codon positions +3 to +10. Calculations are based on the specified codon filling the A site. K-S, Kolmogorov-Smirnov; Lso2, late-annotated short open reading frame 2; WT, wild type.

To gain molecular insight into the translation defects of *lso2Δ*, we analyzed the distributions of ribosome-protected footprints within mRNAs. We captured footprints ranging from 15 to 34 nucleotides in order to examine a broad range of ribosomal complexes, including quality control intermediates (15–18 mers) and those from elongating ribosomes in different states (20–22 mers versus 28–34 mers) [62,72,73]. Analysis of 28–30 mer footprints showed a 5-fold increase in monosomes stalled at the start codon in *lso2Δ* (Fig 5A), which was determined by averaging the expression-normalized ribosome occupancy across all transcripts. Consistently, most genes showed an increased fraction of start codon versus ORF body reads, with a median increase of approximately 4-fold (Fig 5B). This shift lay well outside the variability of start-to-body ratios between biological replicates (plotted as replicate error in Fig 5B). Start codon accumulation was also specific to long footprints, as 20–22 mers did not show higher start codon occupancy (S5A Fig). The magnitude of long footprint accumulation was comparable to the effect of depleting eukaryotic translation initiation factor 5A (eIF5A; median increase of approximately 4-fold, S5B Fig), an essential factor that promotes peptide bond formation through pause-prone codons at the +2 position and elsewhere in the gene body [65,74,75]. However, unlike recovering *lso2Δ*, eIF5A-depleted cells do not accumulate empty monosomes [76], which underscores the functional distinction that Lso2 more broadly affects initiation (Fig 4).

To understand why long versus short footprints accumulate at start codons and why some genes are preferentially affected, we looked for determinants of start pausing. We first compared nucleotide usage at positions −13 to +16 in the top quartile of genes with the largest start pause change in *lso2Δ* versus in all genes. The bias in nucleotide frequency was quantified using the log-likelihood ratio (G-statistic, Materials and methods) [77]. Genes with increased start pausing are strongly biased toward T at +4 and C at +5, with weaker biases downstream and at −9 in the transcript leader (Fig 5C). We then extended this analysis by computing single codon occupancies for all +2 codon identities (Fig 5D). Consistent with the increased +4 T and +5 C representation in *lso2Δ*-sensitive genes, serine codons showed the greatest increase in pausing (Fig 5E and 5F). However, AGC and AGT Ser codons also led to increased pausing (S5C Fig), indicating that the effect of serine is driven by the identity of the amino acid. Additional significant *lso2Δ-*sensitive pauses were seen at proline, glycine, alanine, glutamine, asparagine, aspartate, and threonine +2 codons (Fig 5F). This defect was strongly position-dependent: plotting the change in pause score across the first 10 positions revealed a sharp decline from codon 2 to 3, and in all cases, pausing was attenuated by codon 5 (Fig 5G). Furthermore, pause changes at these codons within ORF bodies were smaller than and uncorrelated with those in the early ORF (S5D Fig). These results suggest that start pausing is unlikely to be caused by decreased tRNA charging, whose effects would also extend to ORF bodies. Instead, the start-paused ribosomes may be slow to form the first peptide bond, generating a footprint size characteristic of this substep of elongation [73]. It is currently unclear whether early pausing and empty 80S accumulation arise from a common mechanism, such as defective 60S joining, or from distinct perturbations. However, the difference between early versus ORF-body behavior suggests that peptide bond formation at some codons is most affected immediately downstream of initiation in *lso2Δ*.

Importantly, we note that the identities of the most affected +2 codons and the specific accumulation of ribosomes at the start codon are distinct from those in eIF5A depletion [65], in which ribosomes accumulate in the broader, approximately 100-codon window beginning at start. Therefore *lso2Δ*’s start-pausing behavior is not a consequence of insufficient eIF5A. Finally, *lso2Δ*-sensitive codons account for 60% of second codons in the genome and 76% in the WT translatome under these growth conditions. Thus, the lack of Lso2 leads to a widespread and codon-specific defect in late initiation or early elongation.

### Absence of Lso2 leads to pervasive changes in global elongation and termination

**Fig 6:**
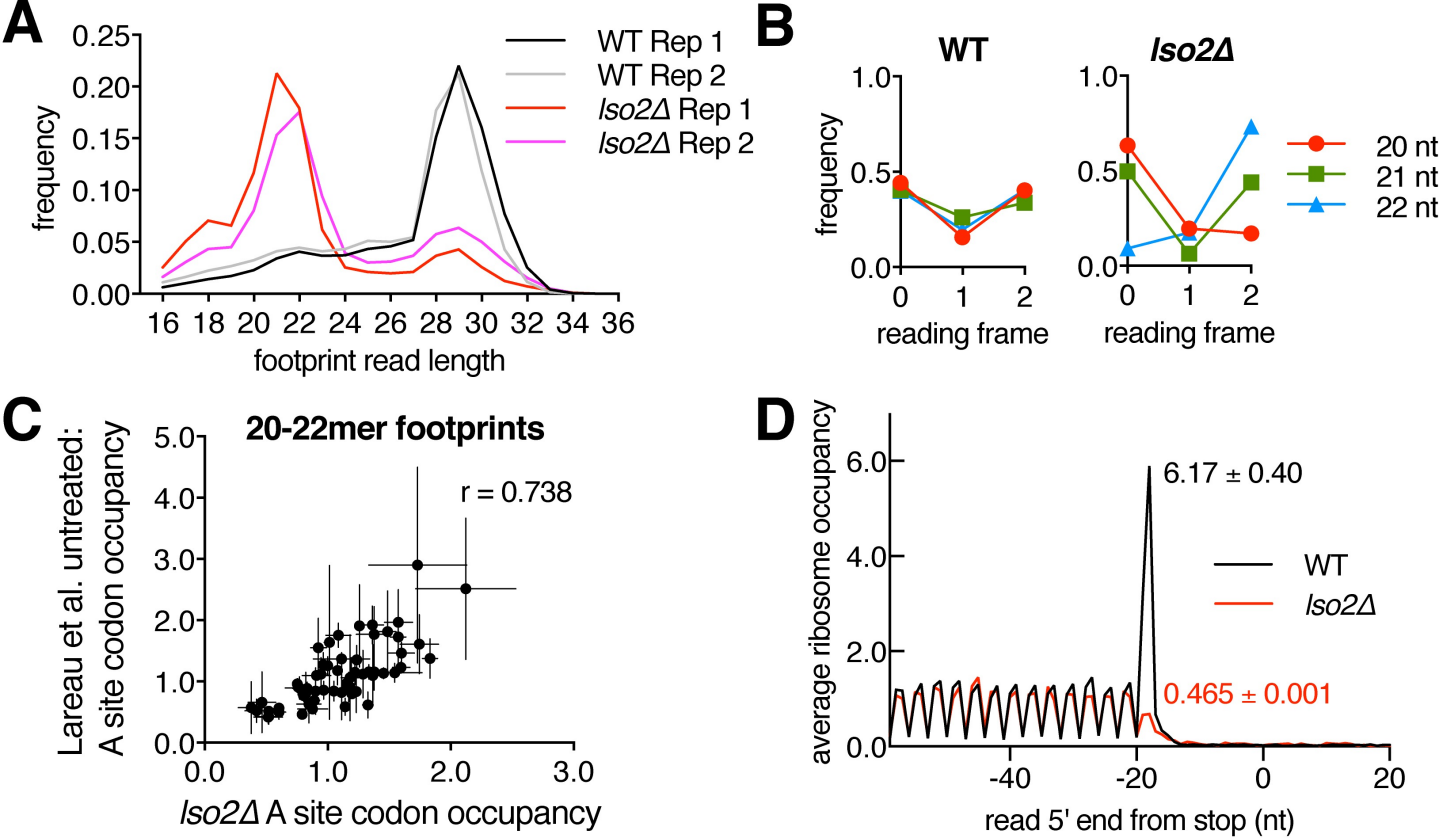
Absence of Lso2 leads to pervasive changes in global elongation and termination. (A) Distribution of footprint read lengths from all CDS regions in WT and *lso2Δ* libraries. (B) Reading frame analysis of 20–22 mer footprints from WT and *lso2Δ* libraries based on 5′ ends. (C) Correlation of 20–22 mer codon occupancies from *lso2Δ* versus 20–22 mer codon occupancies from the WT untreated condition of [73]. Plotted are the means ± S.D. of 2 *lso2Δ* and 3 untreated biological replicates. (D) As in Fig 5A, except that read 5′ end is relative to the stop codon. Numbers indicate the respective stop codon occupancies in WT and *lso2Δ*. See also S5 Fig. CDS, coding sequence; Lso2, late-annotated short open reading frame 2; WT, wild type.

In addition to these perturbations observed in long (28–30 mer) footprints, there was a dramatic shift toward short (20–22 mer) footprints in *lso2Δ* compared to WT (Fig 6A). In contrast to those from WT, short footprints from *lso2Δ* align to a specific frame (Fig 6B). This suggests that a change in translation elongation underlies their production and not merely the absence of Lso2 as a steric barrier to RNases accessing the mRNA channel during footprinting. More specifically, the increased ratio of short versus long footprints in *lso2Δ* suggests a global shift in the distribution of ribosomes between distinct functional states during the elongation cycle. Twenty to 22 mers were previously proposed to originate from rotated ribosomes in the post-peptidyl transfer, pre-translocation state based on footprinting with or without various elongation-inhibiting antibiotics [73]. The codon-specific A site occupancies observed in 20–22 mers from *lso2Δ* were positively correlated with those of 20–22 mers observed in untreated (no cycloheximide) yeast (Pearson r = 0.738, Fig 6C) [73]. This suggests that 20–22 mers in *lso2Δ* arise from an increased fraction of ribosomes in a state that occurs in WT ribosomes. Given that Lso2 is monosome bound and substoichiometric to RPs, this change in ribosome state is unlikely to come from a direct function of Lso2 in modulating every elongation cycle. Instead, because Lso2 binds near the base of the ribosomal stalk, we propose that loss of Lso2 could compromise the function of the stalk in interacting with core elongation factors [78]. This defect may be further compounded by insufficient levels of core elongation factors such as eEF2 (see Discussion).

Finally, ribosome occupancy at stop codons was markedly decreased (Fig 6D), suggesting that termination and recycling proceed more efficiently. Given its 80S enrichment, Lso2 is unlikely to be a direct inhibitor of termination. Instead, assuming that the protein levels of release factors are normal in *lso2Δ*, this result would be consistent with a globally reduced number of translating ribosomes and a higher ratio of release factors to ribosomes at stop codons. Together, these ribosome profiling data indicate that Lso2 is globally required for elongation during this recovery regime.

### Decreased relative translation correlates with start codon pausing in *lso2Δ*

**Fig 7:**
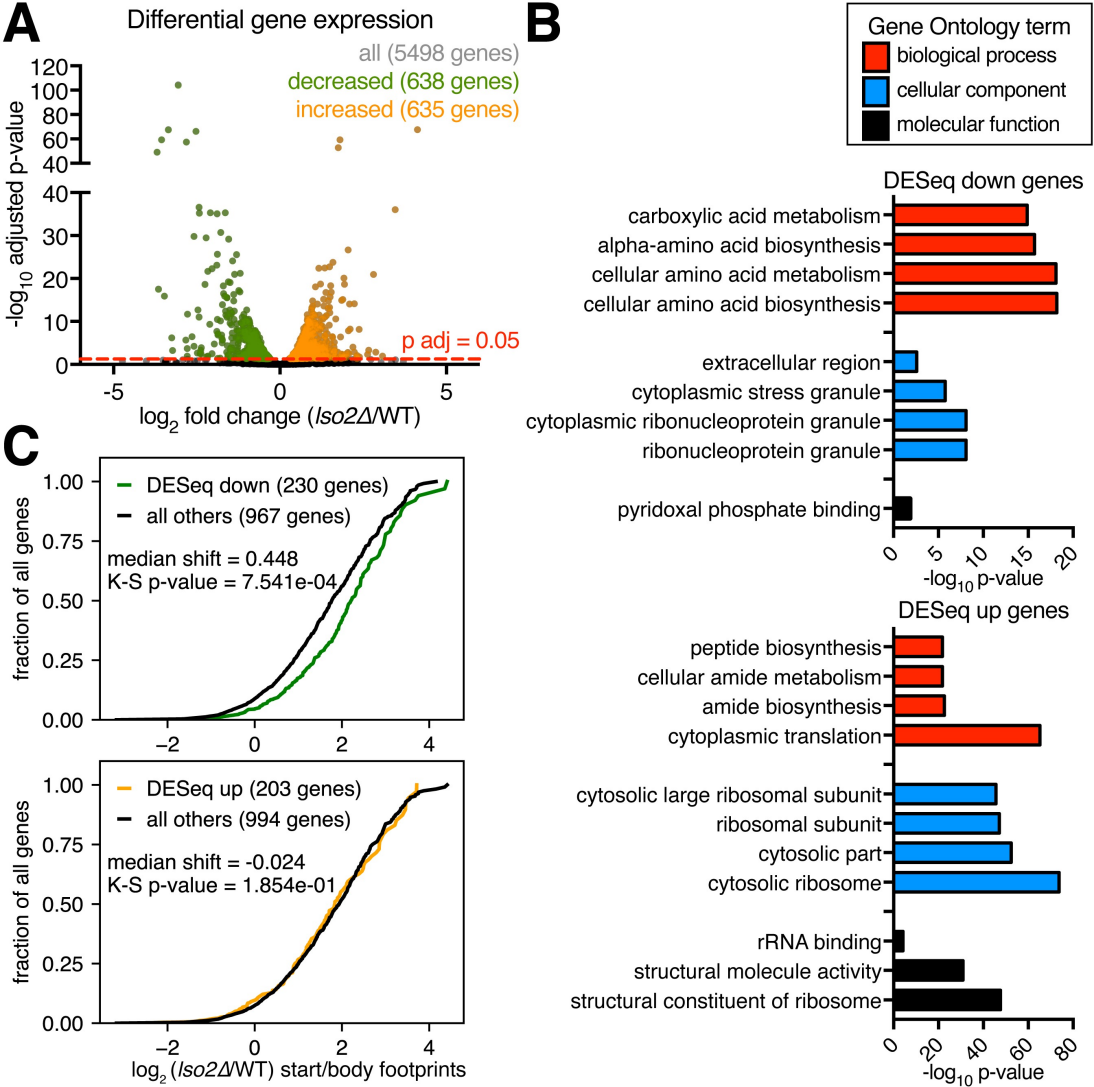
Differential gene expression in *lso2Δ* correlates with start codon pausing. (A) Benjamini-Hochberg-corrected *p*-value versus fold change in footprints for *lso2Δ* versus WT, as determined by DESeq2. Decreased or increased genes with adjusted *p*-value < 0.05 are highlighted. *LSO2* (log_2_ fold change = −9.61, *p*-adjusted = 3.00E-18) is omitted for space. (B) Gene Ontology annotations and *p*-values of enrichment for the DESeq-decreased and DESeq-increased categories, respectively. (C) Empirical cumulative distribution plots of the change in start codon to body read ratio for DESeq-decreased (top) and DESeq-increased (bottom) genes, respectively. Only genes with ≥64 reads were included. See also S6 Fig and S4 Table. K-S, Kolmogorov-Smirnov; rRNA, ribosomal RNA; WT, wild type.

We next asked whether these specific initiation and elongation defects could underlie perturbations to the relative translation of mRNAs in *lso2Δ* versus WT. Differential gene expression analysis of footprint counts (DESeq2) [79] identified 635 genes with (relatively) increased and 638 genes with (relatively) decreased translation (*p*-adjusted < 0.05, Fig 7A) against the backdrop of a global 5-fold decrease in ribosome loading on mRNAs (Fig 4H). These hypersensitive (decreased) and relatively insensitive (increased) gene sets were enriched for distinct Gene Ontology categories (S4 Table): Among the increased set, RP mRNAs drove enrichment of “cytoplasmic translation,” while functions related to amino acid biosynthesis dominated the decreased set (Fig 7B). The down-regulated categories are likely due to strong repression of *GCN4*, the master regulator of amino acid biosynthetic gene transcription (absolute decrease of 20-fold in *lso2Δ*). Indeed, the transcriptional targets of general control nonderepressible protein 4 (Gcn4) [80] are overrepresented among the decreased genes (74/638) versus in the transcriptome (377/5,498 genes, Fisher’s exact test *p* < 0.0001). The log-phase translational efficiency of a gene, which is particularly high for the RP mRNAs, was not predictive of its relative translation in *lso2Δ* (Pearson r = 0.0297, *p* = 5.2E-2). Nor was the presence of an upstream AUG (S6 Fig). Instead, we saw a modest but significant contribution from ribosome pausing at the start codon: DESeq-decreased genes showed higher levels of start codon accumulation in *lso2Δ* (Fig 7C) and were enriched for *lso2Δ*-sensitive +2 codons (Fig 5F) relative to all genes analyzed (Fisher's exact test *p* = 5.7E-3). However, the overall correlation between the change in start codon pausing versus the change in footprints was weak (Pearson r = −0.107), suggesting that it is not the main driver of gene expression levels globally. Unexpectedly, the RP mRNAs also maintained high relative expression despite their overrepresentation of +2 serine codons. We conjecture that other *cis* features promote their translation and/or that their expression is increased as an adaptive response to inadequate ribosome function. In summary, increased pausing at the start codon explains some but not all changes to the relative translation of mRNAs in *lso2Δ*.

## Discussion

Here, we present the discovery and characterization of a conserved ribosome-bound protein that we show is required in yeast for translation during recovery from extended starvation. Through genome-wide identification of RNA crosslinks, we mapped a ribosome binding site for Lso2 to the universally conserved GAC, located within 30 angstroms of the A site on the 60S subunit. Consistent with this location, Lso2 crosslinks to tRNAs in vivo and stabilizes ribosomal subunit association in vitro. Additionally, Lso2’s ribosome-binding activity is conserved between yeast and humans: a subpopulation of human CCDC124 comigrates with 80S ribosomes in HeLa cells, while CCDC124 expressed from the yeast *LSO2* locus interacts with the same region of the 60S subunit. Loss of Lso2 during recovery from extended starvation causes initiation and elongation defects on virtually all genes in the transcriptome. Thus, this study demonstrates the potential to uncover new roles in translation and physiology for uncharacterized ribosome-associated proteins.

Following detection of Lso2 in ribosomal complexes using proteomics (Fig 1B) and in vivo crosslinking (Fig 2B and S2B Fig), we demonstrated its direct interaction with ribosomes using an in vitro approach. Specifically, purified recombinant Lso2 was shown to stabilize the association of empty ribosomal subunits (Fig 2I), which is a known property of small proteins binding in the inter-subunit face. However, we also observed in vivo that the absence of Lso2 during starvation recovery causes an increase in empty 80S monosomes rather than an accumulation of free ribosomal subunits (Fig 4B). This phenotype thus suggests that the cellular function of Lso2 is more complex than its activity toward empty subunits in vitro. We note key functional distinctions between Lso2 and 2 other proteins that also bind in the tRNA channel, the conserved prokaryotic GTPase upstream of Leader peptidase (LepA) and the eukaryotic ribosome preservation factor suppressor of Tom1 (Stm1). Whereas LepA is required for ribosome assembly [81] but only subtly affects global elongation as measured by ribosome profiling [82], elongation in gene bodies is severely perturbed in recovering *lso2Δ* (Fig 6A). Lso2 is also not required for maintaining bulk levels of cellular ribosomes during stationary phase (S4H Fig), a function previously demonstrated for Stm1 [60].

Using a refined protocol for ribosome profiling (without drug treatment and with internal standards), we comprehensively characterized the 80S accumulation and other defects in recovering *lso2Δ* (Figs 4F–4H and 5–7). These include a 5-fold decrease in global initiation, a 4-fold accumulation of long footprints from translating monosomes stalled at a subset of start codons, and a strong shift in footprint read lengths from long (28–34 nt) to short (20–22 nt). Given the binding site of Lso2, we suggest a mechanism whereby compromised interaction of GTPases with the GAC underlies perturbations to multiple stages of the translation cycle (see below).

It has previously been observed that removing rRNA or protein components of the GAC reduces ribosome binding and the GTP hydrolysis rates of initiation and elongation factors in vitro [83,84]. The *lso2Δ* ribosome profiling signature parallels these defects. The reduced number of mRNA-bound ribosomes and increase in start-paused monosomes could reflect dual consequences of a failure to correctly join 60S subunits to the 43S preinitiation complex, which requires the GTPase eukaryotic translation initiation factor 5B (eIF5B) [85,86]. Whereas failure to recruit eIF5B generally leads to destabilized 43S and an accumulation of empty ribosomes [85], the 80S that do engage on mRNAs may be impaired in transitioning to elongation. Here, the bottleneck could arise from slow eIF5B GTP hydrolysis and dissociation [86,87]. It is also conceivable that defective initiation further disrupts the first round of peptide bond formation, which is typically slow because of the absence of a rigidifying nascent chain [88]. In support of this latter hypothesis, the magnitude of start pausing varied according to +2 amino acid identity, with codons for small and polar amino acids being more challenging to translate in *lso2Δ* (Fig 5F). In addition to these initiation and early elongation defects, broad changes to codon occupancies in ORF bodies (S5D Fig) and the strong accumulation of 20–22 mer footprints (Fig 6A) also indicate perturbed general elongation. The high correlation between 20–22 mer codon occupancies in *lso2Δ* and those observed in untreated WT cells (Fig 6C) [73] suggests that *lso2Δ* ribosomes spend uniformly more time in an elongation state found in WT cells. The initial proposal of [73] points to decreased eEF2 recruitment as one explanation. Alternatively, changes in association of other core elongation factors could also affect elongation speed and the relative abundance of different footprint lengths. Finally, these primary defects may lead to insufficient synthesis of new translation factors during recovery, which would further compound their magnitude.

How might Lso2 affect the function of the GAC? Given that Lso2 is monosome enriched and only one-tenth the abundance of all ribosomes, any functional model must rationalize the magnitude and persistence of *lso2Δ* defects throughout translation without invoking direct interaction with all ribosomes. We consider that its rRNA crosslink cluster is located at the base of the ribosomal stalk and directly contacts the essential RP P0. P0 in turn scaffolds interaction with the heterodimers P1A/B and P2A/B, the lateral constituents of the stalk [89,90]. Together, this pentameric complex recruits translational GTPases including eIF5B, eEF1, and eEF2 [91,92]. We tested whether loss of Lso2 reduces the stoichiometry of P-stalk proteins during starvation recovery; however, their 80S ribosome occupancy was unaffected (S7 Fig), suggesting that Lso2 affects GAC function other than via binding of its protein constituents. The increased sensitivity of *lso2Δ* 60S subunits to RNase digestion (S4I Fig) raises the possibility of structural changes to the GAC. How these changes might affect GAC interaction with different elongation factors awaits further investigation.

Although Lso2 is constitutively expressed, it is only required for global translation during starvation recovery (Fig 4A and S4A Fig). One explanation for this condition-specific role is metabolic shifts that cause biochemical changes to the ribosome. Quiescent cells rely on oxidative phosphorylation for their energy requirements [59,93], which leads to an increase in free radicals that can oxidize RNA. Furthermore, quiescent cells show increased proteolytic activity [94] and have degraded bulk cellular ribosomes to a small fraction of log-phase levels [60,94]. This increased catabolism could also damage the structure and function of the remaining ribosome pool. However, the low ratio of Lso2 to ribosomes during starvation recovery also suggests that any protective function must be dynamic as opposed to stoichiometric binding by the classic ribosome preservation factors.

Given that the GAC is structurally conserved across kingdoms and that CCDC124 and Lso2 crosslink to the same location on yeast ribosomes, a subset of CCDC124 likely also binds the GAC of human ribosomes. It remains to be determined in human cells whether CCDC124 is required for efficient initiation and elongation under conditions that challenge the integrity of the GAC, a function that would justify the conservation of putative Lso2 orthologs throughout eukaryotes.

## Materials and methods

### Yeast strains and culture conditions

All experiments except for purification of ribosomal subunits were conducted in the Sigma1278b, Strain F1950 background (*MATa ura3 his3 leu2 trp1*, gift of Dr. Hiten Madhani) [95]. Ribosomal subunits were purified from *lso2Δ* in BY4742 (*MATα his3Δ1 leu2Δ0 lys2Δ0 ura3Δ0 lso2Δ*::*kan*).

Strains were cultured in liquid YPAD (1% yeast extract, 2% peptone, 2% glucose, 0.01% adenine hemisulfate) or on solid YPAD agar (2%). For exponential phase, strains were grown at 30°C with shaking at 200 rpm from an initial OD_600_ ≤ 0.15 to a final OD_600_ of 1.0. For glucose starvation, exponential cultures at OD_600_ 1.0 were spun down and resuspended in prewarmed YPA (lacking glucose) and then grown with shaking at 30°C for the indicated times. To assay recovery from stationary phase, strains were first grown for 96 hours from OD_600_ 0.05 to approximately OD_600_ 8, with both WT and *lso2Δ* saturating around 48 hours. Four hundred OD_600_ units of cells (about 50 mL of saturated culture) were then spun down and resuspended in 1.0 L of prewarmed fresh YPAD for a final concentration of OD_600_ 0.4. Recovery cultures were grown at 30°C with shaking at 200 rpm.

### Yeast strain construction

For Myc-tagged strains, the 9xHis-2xPreScission Protease Site-9xMyc-*HIS3* cassette in the pJS-HPM53H plasmid [96] was amplified with homology to replace the stop codon of the gene to be tagged (see S5 Table for primer sequences). Strains were confirmed by sequencing across the entire fusion gene and western blotting for the Myc epitope.

V5-tagged strains were constructed by 2-step markerless replacement. In the first step, the *LSO2* CDS was replaced by the *URA3* CDS via transformation of a homology-flanked PCR cassette, which was amplified from genomic DNA using OYW95 and OYW96. Transformants were then selected on minimal media lacking uracil. In the second step, the *URA3* CDS was replaced by transformation of a homology-flanked PCR cassette bearing the fusion gene of interest. Ura^−^ transformants were then selected on 5-fluorootic acid. The *CCDC124* CDS was ordered as a synthetic gene from Integrated DNA Technologies with a yeast codon-optimized nucleotide sequence (S5 Table). *LSO2-* and *CCDC124-5XGly-V5* cassettes were amplified in pieces and stitched together by assembly PCR, with primers adding in the linker and epitope tag sequences (S5 Table). Strains were confirmed by sequencing of the fused tag and western blotting for the V5 epitope.

Single mutants were constructed by transformation of a PCR cassette containing 40 base pairs of homology to either side of the targeted region [97]. For *lso2Δ*::*kan* deletion strains, the kanMX6 cassette in pFA6a-kanMX6 was amplified with homology to replace the *LSO2* CDS using OWG801 and OWG802. All deletion strains were confirmed by PCR genotyping of the target locus using OWG803 (−200 of the start codon) and OYW98 (+200 of the stop codon).

### Yeast gradient profiling

Cycloheximide (Sigma C7698) was added to growing culture to a final concentration of 0.1 mg/mL and shaken for 2 minutes. Cultures were quickly poured over ice, spun down, and washed twice with ice-cold Polysome Lysis Buffer (PLB; 20 mM HEPES-KOH pH 7.4, 2 mM MgAc, 0.1 M KAc, 0.1 mg/mL cycloheximide, 1% TritonX-100). For lysis, cells were vortexed with glass beads and PLB supplemented with 1 mM PMSF and 1X EDTA-free protease inhibitors (Roche 11836170001). Extracts were clarified at 21,000 × *g* for 20 minutes.

For absorbance profiling in 2 mM Mg, 10–25 OD_260_ units were loaded onto 10%–50% sucrose PLB gradients, followed by centrifugation at 35,000 rpm in a Beckman SW41 rotor for 3 hours. For gradient profiling in high salt, 4 M KCl was added to cell extract (10 OD_260_ units) immediately prior to centrifugation to raise the final concentration to 0.8 M KCl. Extract was then loaded onto 10%–40% sucrose PLB gradients containing 0.8 M KCl and centrifuged at 35,000 rpm in a Beckman SW41 rotor for 3 hours. For gradient profiling in EDTA, 60 OD_260_ units of extract made with PLB were loaded onto 10%–30% sucrose PLB gradients containing 25 mM EDTA. Gradients were centrifuged at 18,000 rpm in a Beckman SW28 rotor for 16 hours. All gradients were fractionated from the top down using a Biocomp Gradient Station (Biocomp Instruments) with continual monitoring of absorbance at 254 nm.

### Mass spectrometry of ribosomal complexes

Replete or 2 hours glucose-starved cultures were harvested and extracts prepared in PLB as described in “Yeast gradient profiling.” For the first round of polysome isolation, 200 OD_260_ units of each sample were loaded onto a 10%–50% sucrose PLB gradient and centrifuged in a Beckman SW28 rotor at 28,000 rpm for 4 hours. Gradients were fractionated on a Biocomp gradient station and the polysome region collected manually. Polyribosomes were pelleted through a sucrose cushion (20 mM Hepes-KOH pH 7.4, 10 mM MgAc, 0.5 M KAc, 1.0 M sucrose, 2 mM DTT) and the ribosome pellet resuspended in subunit separation buffer lacking DTT (50 mM HEPES-KOH pH 7.4, 5 mM MgCl_2_, 0.5 M KCl) to a final concentration of about 50 OD_260_ units per mL. One unit of RNase I per OD_260_ unit was added and the mixtures incubated at 24°C for 30 minutes. RNase digest mixtures were immediately loaded onto 10%–50% sucrose subunit separation buffer gradients and centrifuged in a Beckman SW28 rotor at 18,000 rpm for 16 hours. The 40S, 60S, and 80S fractions were pooled and then concentrated and exchanged into 0.1 M ammonium bicarbonate (pH 8.0). Total protein concentrations were determined by the Bradford assay.

Downstream processing for mass spectrometry was as follows. Proteins were digested in parallel reactions using trypsin and lysC, respectively. Prior to trypsin digestion, peptides were partially modified with propionic anhydride to block lysines and thereby produce longer peptides in an attempt to increase sequence coverage. Peptide digests were differentially labeled with mTRAQ nonisobaric amine reagents (mTRAQ*Δ*0 and mTRAQ*Δ*8, Sciex 4440015 and 4427700). The labels were flipped between biological replicates. Labeled peptides from each condition were mixed 1:1 based on the total protein concentration and then fractionated by off-gel electrophoresis to decrease sample complexity prior to analysis by MS/MS.

Mass spectrometry followed the method of [98] with modifications. All peptide samples were separated on an online nanoflow HPLC system (Agilent 1200) and analyzed on an LTQ Orbitrap Velos mass spectrometer (Thermo Fisher Scientific). Approximately 1 μg of sample was injected onto a fused-silica capillary column (New Objective, PicoFrit PF360-75-10-N-5 with 10 μm tip opening and 75 μm inner diameter) packed in-house with 14 cm of reversed phase media (3 μm ReproSil-Pur C18-AQ media, Dr. Maisch GmbH). The HPLC setup was connected via a custom-made nanoelectrospray ion source to the mass spectrometer. After sample injection, peptides were separated at an analytical flowrate of 200 nL/min with a 70-minute linear gradient (about 0.29%B/min) from 10% solvent A (0.1% formic acid in water) to 30% solvent B (0.1% formic acid/90% acetonitrile). The run time was 140 minutes for a single sample, including sample loading and column reconditioning. MS1 spectra were acquired in the Orbitrap with a resolution of 60,000 and a mass range from 300 to 1,800 m/z. For CID scans, collision energy was set to 30; for ETD scans, the reaction time was 50 ms. The maximum inject time was 50 ms, and maximum ion count was 1 × 10^4^ counts. mTRAQ samples were analyzed with Top12 methods. We used an isolation window of 3 Th for isolation of ions prior to MS/MS. Ions selected for MS/MS were dynamically excluded for 20 seconds after fragmentation.

### Quantification and identification of peptides and proteins

Mass spectrometry data were analyzed by the methods of [98–100] with modifications. All mass spectra were processed using the Spectrum Mill software package v4.0 beta (Agilent Technologies), which includes a module developed by us for iTRAQ and mTRAQ-based quantification. Precursor ion quantification was done using extracted ion chromatograms (XICs) for each precursor ion. The peak area for the XIC of each precursor ion subjected to MS/MS was calculated automatically by the Spectrum Mill software in the intervening high-resolution MS1 scans of the LC-MS/MS runs using narrow windows around each individual member of the isotope cluster. Peak widths in both the time and *m*/*z* domains were dynamically determined based on MS scan resolution, precursor charge, and *m*/*z*, subject to quality metrics on the relative distribution of the peaks in the isotope cluster versus theoretical. Similar MS/MS spectra acquired on the same precursor *m/z* in the same dissociation mode within +/− 60 seconds were merged. MS/MS spectra with precursor charge >7 and poor-quality MS/MS spectra, which failed the quality filter by not having a sequence tag length >1 (i.e., minimum of 3 masses separated by the in-chain mass of an amino acid), were excluded from searching.

For peptide identification, MS/MS spectra were searched against protein sequences from reference Sigma1278b MIT_2009_ACVY01000000 on *Saccharomyces* Genome Database. All ORFs were included except the following: those with ≥1 N, those whose CDS length was not divisible by 3, those that did not begin with methionine, those that did not contain a stop codon at the end, and those that contained ≥1 stop codon. For each of these cases except the presence of ≥1 N, the S288C protein sequence was substituted unless it was also erroneous. Search parameters included ESI–Linear-Ion-Trap (CID or ETD) scoring parameters, trypsin or LysC enzyme specificity with a maximum of 3 missed cleavages and KP or RP cleavages allowed, +/− 20 ppm precursor mass tolerance, +/−0.7 Da product mass tolerance, and carbamidomethylation of cysteines and mTRAQ labeling of lysines and peptide n-termini as fixed modifications. Allowed variable modifications were oxidation of methionine and phosphorylation of serine, threonine, or tyrosine residues with a precursor MH+ shift range of −18 to 177 Da. For propionylated samples, propionylation of lysine was searched as a fixed modification. Identities interpreted for individual spectra were automatically designated as valid by optimizing score and delta rank1-rank2 score thresholds separately for each precursor charge state in each LC-MS/MS while allowing a maximum target decoy–based false discovery rate (FDR) of 1.0% at the spectrum level. This yielded a final overall FDR at the peptide level of <1%.

In calculating scores at the protein level and reporting the identified proteins, redundancy was addressed in the following manner: The protein score is the sum of the scores of distinct peptides. A distinct peptide is the single highest scoring instance of a peptide detected through an MS/MS spectrum. MS/MS spectra for a particular peptide may have been recorded multiple times (i.e., as different precursor charge states, modified by oxidation of Met) but are still counted as a single distinct peptide. When a peptide sequence >8 residues long is contained in multiple protein entries in the sequence database, the proteins are grouped together, and the highest-scoring one and its accession number are reported. In some cases when the protein sequences are grouped in this manner, there are distinct peptides that uniquely represent a lower scoring member of the group (isoforms or family members). Each of these instances spawns a subgroup, and multiple subgroups are reported and counted toward the total number of proteins.

mTRAQ ratios were obtained from the protein summary details export table in Spectrum Mill. To obtain mTRAQ protein ratios, the median was calculated over all distinct peptides assigned to a protein subgroup in each biological replicate.

We note that a common peptide was assigned to both Sdd3 and Ygr110w and used to compute their mTRAQ ratios. Given that Ygr110w is the bona fide mitochondrial protein cardiolipin-specific deacylase 1 (Cld1) and was previously shown to localize exclusively to mitochondria [101], we subsequently validated Sdd3 as a starvation-enriched ribosome-bound protein.

### Lso1 induction

A Myc-tagged *LSO1* strain was grown from OD_600_ 0.1 to 1.0 in YPAD supplemented with the indicated concentration of copper sulfate (Sigma 209198). During the same period, the medium was also supplemented with either 1-hydroxypyridine-2-thione zinc (Sigma H6377), neocuproine hydrate (Sigma 121908), or the corresponding volume of vehicle (DMSO). Whole-cell extracts were made by TCA precipitation and then probed for the Myc epitope and Pgk1 as a loading control, as described in “Western blotting”.

### ePAR-CLIP library preparation

Tagged and untagged strains were grown in synthetic complete medium with 120 μM uracil to OD_600_ 1.0. Then, 4-thiouracil (Sigma 440736) was added to each culture to a final concentration of 500 μM, and cultures were grown with shaking at 30°C for an additional 3 hours. For UV crosslinking, cultures were poured over ice and pelleted and then resuspended in cold water and transferred to a petri dish situated on ice. Resuspended cells were crosslinked in a Stratalinker at 365 nm, approximately 5 cm from the UV bulb, for a total of 7.2 J/cm^2^ (680 seconds). UV-treated culture was pelleted. For eCLIP treatment followed by gradient fractionation to visualize the migration of Lso2-Myc, cycloheximide was added to 0.1 mg/mL following UV crosslinking. Cells were then pelleted.

Cells were lysed by vortexing with glass beads in lysis buffer (50 mM Tris-HCl pH 7.4, 100 mM NaCl, 1% NP-40, 0.1% SDS, 0.5% sodium deoxycholate, 1:200 Protease Inhibitor Cocktail III, 0.44 U/mL murine RNase inhibitor). Extracts were clarified at 10,000 × *g* for 5 minutes.

Downstream library preparation followed the method of [44] with the following modifications. Anti-Myc antibody (10 μg, Sigma M4439) was bound to 100 μL of Dynabeads Protein G (ThermoFisher Scientific 10004D). For RNase digestion, 26 OD_600_ units were diluted in lysis buffer to a final volume of 1.0 mL in lysis buffer. RNase I was added to a final concentration of either 1:20,000,000 (set 1: Fig 2B and 2D left, and S2B Fig) or 1:2,000,000 (set 2: Fig 2D right, 2E–2G, S2E Fig, S3C and S3D Fig; S2 and S3 Tables) and incubated with extracts for 15 minutes at 22°C and shaking at 1,200 rpm. IP with Myc-coupled beads proceeded for 2.5 hours at 4°C. For 3′ adapter ligation, samples were ligated to pre-adenylated adapter OJA225 (5′-5Phos/TGGAATTCTCGGGTGCCAAGG/3ddC/) using T4 RNA Ligase 1 (NEB M0437M). As a diagnostic of crosslinking efficiency and approximate RNA size distribution, 10% of each IP and 10% of each untagged sample were removed for [γ-^32^P]ATP labeling and then resolved by SDS-PAGE on a 4%–12% Bis-Tris gel. Radiolabeled RNPs were transferred from the gel to a PVDF membrane, which was exposed to a phosphorimager screen to produce the images in Fig 2D, S2D Fig, and S3C Fig. Input (0.5%) and IP (13%) were separated by SDS-PAGE and analyzed by western blotting for the Myc epitope. The remainder of each sample was loaded on a different gel, separated by SDS-PAGE, and transferred to a PVDF membrane. For the first set (made with 1:20,000,000 RNase I), the region of the membrane from 55 to approximately 130 kDa was excised for all samples to avoid a nonspecific species migrating around 50 kDa in the IP and the untagged libraries. For the second set (made with 1:2,000,000 RNase I), which included CCDC124-Myc, the region of the membrane from 35 to 100 kDa was excised for Lso2-Myc, from 55 to 130 kDa for CCDC124-Myc, from 35 to 130 kDa for their SMI, and from 55 to 130 kDa for the untagged. Primer OWG915 (5′-GCCTTGGCACCCGAGAATTCC) was used for first-strand cDNA synthesis. The barcoded adapter OWG920 (5′-/5Phos/N_10_GATCGTCGGACTGTAGAACTCTGAACGTG/3SpC3/-3′) was used for ligation to the 3′ end of first-strand cDNA. For PCR amplification, OBC (5′-CAAGCAGAAGACGGCATACGAGATN_6_GTGACTGGAGTTCCTTGGCACCCGAGAATTCCA) was used as the forward primer, in which the N_6_ was used to barcode each library on a flow cell, and Illumina RP1 (5′-AATGATACGGCGACCACCGAGATCTACACGTTCAGAGTTCTACAGTCCGA) was used as the reverse primer. Libraries generally required 8–14 cycles of PCR amplification. Paired-end sequencing was performed on an Illumina NextSeq 500 instrument.

Pus1-HPM IP and strain-matched untagged samples were prepared as above, with RNase I added to a final concentration of 1:20,000,000.

### eCLIP read processing and quantification

ePAR-CLIP read processing followed the pipeline of [44] (Supplementary Protocol 2) with the following modifications. Individual libraries were first demultiplexed by their library-level barcodes. For reverse reads, which contained at the first 10 positions a barcode for collapsing PCR duplicates within a library, this decamer was removed and appended to the fastq header. Two rounds of adapter removal using Cutadapt [102] were run with previously published parameters except for the adapter sequences, which were modified to remove OBC from the 5′ side and NNNNNGATCGTCGGACTGTAGAACTCTGAACGTGTAG (embedded in OWG920) from the 3′ side. For mapping, the genome assembly Sigma1278b_MIT_2009_ACVY01000000 was downloaded from *Saccharomyces* Genome Database. To consolidate reads and downstream peak calling on multicopy genes, the genome was modified as follows. All rRNA and tRNA genes containing a 40-base-pair window that multimapped elsewhere in the genome were first masked from the endogenous genome. Single copies of unique tRNA and rRNA genes were then placed on a separate artificial chromosome, with each locus separated by a spacer of 25 Ns. Forward and reverse reads were mapped using STAR [103] with published parameters except that a multimap cutoff of 10 and a mismatch rate of 9% were used. PCR duplicates were collapsed, the mapped files were sorted, and the reverse reads were used for peak identification as described. No replicates were merged in any analyses.

In the first set of libraries (low RNase), 2 Lso2-Myc replicates were used to prepare IP libraries. RNA from each of these replicates was used to prepare paired SMI libraries, and 2 untagged strains (YWG25) were used to prepare untagged libraries. After peak identification using CLIPper, the read fraction in each peak of an IP library was normalized by the read fraction of the corresponding region in the control libraries (SMI and untagged, respectively). Peaks with fold-enrichment >4.0 and *p* < 10^−5^, relative to both controls, and that overlapped by at least 1 nucleotide between the 2 IP replicates, were taken as reproducible targets.

In the second set of libraries (high RNase), 2 Lso2-Myc replicates and 2 CCDC124-Myc replicates were used to prepare IP libraries. One CCDC124-Myc replicate was discarded because of very low RNA recovery. RNA from an Lso2-Myc replicate was used to prepare a SMI library, and YWG25 was used to prepare an untagged library. Because most tRNA genes had <64 uniquely mapping reads, multimapping reads were fractionally assigned to all mapped positions based on the STAR-generated MAPQ score, with the following scaling factors: {0: 0.2, 1: 0.286, 3: 0.5, 255: 1}. Genes with <64 counts thus assigned (as determined by read 5′ ends) were excluded from further analysis. Coverage at each position was normalized by the lengths of the originating reads. The read density of a tRNA gene was then taken as the sum of normalized coverage across all positions in the gene, divided by the total number of primary alignments in that library. We defined enriched tRNA targets as those with a ≥4-fold ratio of read density in both IPs relative to the SMI and untagged.

Mutational analysis of T to C transitions was performed by extracting all rRNA mapping reads using samtools and then following the method of [104]. Outputs are available as supplemental files on GEO under accession GSE109343.

### Purification of recombinant Lso2 from *E*. *coli*

The yeast *LSO2* coding sequence was cloned into pET-TEV-28a(+) using Gibson assembly. pET-TEV-28a(+) is identical to pET-28a(+) (Novagen) except that the thrombin cleavage site is replaced by a tobacco etch virus (TEV) protease cleavage site. OYW50 and OYW51 were used to amplify the *LSO2* CDS from yeast genomic DNA (YWG25). OYW46 and OYW47 were used to linearize pET-TEV-28a(+) with the NheI and the EcoRI sites as the 5′ and 3′ ends, respectively. Gibson assembly was performed according to the manufacturer’s instructions. Correct cassette insertion was confirmed by sequencing. The resulting Lso2 protein is tagged with 6XHis at its N-terminus followed by the TEV protease cleavage site. The plasmid was transformed into *E*. *coli* BL21(DE3) (NEB C2527) for overexpression.

Transformed cells were grown in LB kanamycin from OD_600_ 0.1 to 0.6 at 37°C with shaking. Cultures were then transferred to 18°C and shaken for 30 minutes before inducing with 0.5 mM IPTG (Sigma I6758). Cultures were grown for an additional 22 hours at 18°C before harvesting. Cells were rapidly spun down and washed once with lysis buffer (20 mM HEPES-KOH pH 7.4, 0.1 M KCl, 10% glycerol, 0.1% TritonX-100, 20 mM imidazole).

Cells were lysed by vortexing with glass beads in lysis buffer supplemented with 2X EDTA-free protease inhibitors, 0.1 mM PMSF, and 10 mM β-mercaptoethanol. DNase I (NEB M0303S) was added to extract to a concentration of 10 U per 1 OD_600_ unit of cells, supplemented with 1 M MgCl_2_ to a final concentration of 2.5 mM Mg^2+^. Digestions proceeded at 4°C for 2.5 hours before adding 4 M KCl to raise the final concentration to 0.5 M. Extracts were clarified at 21,000 × *g* for 20 minutes and passed through a 0.2 μm filter before loading onto a nickel sepharose column (HisTrap HP, GE 17524701). The column was washed with 10 volumes of lysis buffer containing 0.5 M KCl and then eluted in a single step using nickel elution buffer (20 mM HEPES-KOH pH 7.4, 0.1 M KCl, 10% glycerol, 0.1% TritonX-100, 250 mM imidazole, 10 mM β-mercaptoethanol). Fractions containing Lso2 were pooled and loaded onto a size-exclusion column (Superdex 200 prep grade, GE 28989335). Lso2 was eluted with 1 column volume of storage buffer (20 mM HEPES-KOH pH 7.4, 0.1 M KCl, 10% glycerol, 2 mM DTT) and concentrated for storage at −80°C.

### Purification of ribosome subunits from yeast

*lso2Δ* in BY4742 was grown at 30°C with shaking to OD_600_ 1.0. Cells were quickly spun down and lysed by vortexing with glass beads in lysis buffer (20 mM HEPES-KOH pH 7.4, 10 mM MgAc, 0.5 M KAc, 1 mg/mL heparin, 2 mM DTT, 1X EDTA-free protease inhibitors). Extracts were clarified at 21,000 × *g* for 20 minutes. The supernatant was then applied to sucrose cushions (20 mM HEPES-KOH pH 7.4, 10 mM MgAc, 0.5 M KAc, 1.0 M sucrose, 2 mM DTT) and centrifuged in a Beckman Type 70 Ti rotor for 106 minutes at 60,000 rpm. The crude ribosomal pellet from this spin was resuspended in subunit separation buffer (50 mM HEPES-KOH pH 7.4, 2 mM MgCl_2_, 0.5 M KCl, 2 mM DTT). Resuspended ribosomes were diluted to a concentration of 200 OD_260_ units per mL in subunit separation buffer. Puromycin (Sigma P7255) was added to a final concentration of 1 mM. Samples were incubated on ice for 15 minutes, followed by incubation at 37°C for 10 minutes. Two hundred OD_260_ units then were layered onto each 5%–20% sucrose gradient (50 mM HEPES-KOH pH 7.4, 5 mM MgCl_2_, 0.5 M KCl, 0.1 mM EDTA, 2 mM DTT) and centrifuged in a Beckman SW28 rotor for 9 hours at 24,000 rpm. Gradients were fractionated as described in “Yeast gradient profiling.” Fractions for each subunit were pooled and then concentrated and exchanged into storage buffer (20 mM HEPES-KOH pH 7.4, 2.5 mM MgAc, 0.1 M KAc, 250 mM sucrose, 2 mM DTT). There was approximately 10% contamination of 40S subunits in the 60S prep due to fraction bleed-through.

### Gradient association assay

For the extinction coefficients of the 40S and 60S subunits, 2.0E7 M^−1^ cm^−1^ and 4.0E7 M^−1^ cm^−1^ were used, respectively [105]. An extinction coefficient of 5,500 M^−1^ cm^−1^ was used for recombinant Lso2, based on the prediction formula of [106]. One micromolar (100 pmol) each of 40S and 60S subunits was mixed in buffer E with 3 mM magnesium (20 mM Tris-HCl pH 7.5, 3 mM MgAc, 0.1 M KAc pH 7.6, 2 mM DTT, 0.25 mM spermidine). We note that in practice, there was a small stoichiometric excess of 40S subunits due to incomplete 40S/60S separation during subunit purification (see above). One micromolar of Lso2 or the equivalent amount of buffer was then added to the subunit mix, with the final volume of Lso2 storage buffer (which lacks magnesium) not exceeding 0.7%. The mixture was then incubated at 37°C for 10 minutes, snap cooled on ice, and loaded onto 10%–30% sucrose PLB gradients with 3 mM MgAc. Gradients were centrifuged in a Beckman SW28 rotor at 18,000 rpm for 16 hours. Fractionation was performed as described in “Yeast gradient profiling”.

### Quantification of gradient profiles

For quantification of polysome-to-monosome ratios, the baseline was first defined as the global minimum excluding the manually selected free RNP peak. The 80S_start_ boundary was manually selected from the trough between the 60S peak and 80S peak, and the 80S_end_ boundary was manually selected from the trough between the 80S peak and the disome peak. The monosome area was calculated by Riemann summation between the 80S_start_ and 80S_end_ boundaries, and the polysome area was calculated by Riemann summation between 80S_end_ and the final point in the profile. The areas were divided to obtain the ratio.

For quantification of monosome to subunit ratios, the baseline was first defined as the global minimum. The 40S_start,_ 60S_end_, and 80S_end_ boundaries were manually selected. The monosome area was calculated by Riemann summation between 60S_end_ and 80S_end_ boundaries, while the subunit area was calculated by Riemann summation between 40S_start_ and 60S_end_. The areas were divided to obtain the ratio.

### HeLa cell gradient profiling

HeLa cells (human cervix adenocarcinoma; CCL-2, ATCC) were cultured in DMEM (Sigma D6429) supplemented with 10% fetal bovine serum (Atlanta Biologicals S11150) at 37°C and 5% CO_2_. Cells were collected by scraping and pelleting and then syringe lysed in 2X lysis buffer (20 mM Tris-HCl pH 7.5, 10 mM MgCl_2_, 0.2 M KCl, 1% TritonX-100, 0.2 mg/mL cycloheximide, 4 mM DTT, 2X EDTA-free protease inhibitors) for polysome profiling or in 2X lysis buffer with 2 mM EDTA and lacking MgCl_2_ for EDTA dissociation profiling. Debris and nuclei were pelleted at 1,300 × *g* for 10 minutes. For polysome profiling, 5 OD_260_ units were loaded onto each 10%–50% sucrose gradient (20 mM HEPES-KOH pH 7.4, 5 mM MgCl_2_, 0.1 M KCl, 2 mM DTT, 0.1 mg/mL cycloheximide) and centrifuged in a Beckman SW41 rotor for 3 hours at 35,000 rpm. For EDTA dissociation profiling, 10 OD_260_ units were loaded onto each 10%–30% sucrose gradient (20 mM HEPES-KOH pH 7.4, 0.1M KCl, 2mM DTT, 0.1 mg/mL cycloheximide, 2 mM EDTA). Gradients were centrifuged in a Beckman SW41 rotor at 18,000 rpm for 16 hours. Gradients were fractionated as described in “Yeast gradient profiling”.

### Western blotting

As part of eCLIP, samples were mixed with 3X Laemmli buffer and boiled before loading. For yeast extract gradient fractions, each fraction from a gradient was mixed with 3X Laemmli buffer, boiled, and loaded in equal volume. For quantification of Lso2-V5/Asc1 ratios in exponential phase versus stationary phase recovery, yeast extracts were mixed with 3X Laemmli buffer, diluted in a 2-fold series, and boiled. For HeLa extract gradient fractions, equal volumes of gradient fractions were first removed and diluted to no more than 15% sucrose and then precipitated in 20% TCA with 0.8 mg/mL sodium deoxycholate as a coprecipitant. Each pellet was resuspended in 1X Laemmli buffer and boiled before loading.

Protein samples were separated by SDS-PAGE on Bis/Tris or Tris/glycine gels. Proteins were transferred onto PVDF membranes for eCLIP and nitrocellulose membranes for all other experiments. Membranes were blocked in 5% nonfat milk and then incubated with primary antibody overnight in high-salt TBST (10 mM Tris-HCl pH 7.5, 0.1% Tween, 0.5 M NaCl). The following primary antibodies and concentrations were used: 1:5K anti-c-Myc (Sigma M4439), 1:2.5K anti-Pgk1 (Life Technologies 22C5D8), 1:10K anti-Asc1 [107], 1:5K anti-V5 (Sigma V8137), 1:1K anti-RPS5 (Abcam ab168823), 1:5K anti-CCDC124 (Abcam ab184771), 1:10K anti-Rpl8 (custom-raised in rabbit), 1:500 anti-Rpp0 (MBL International RN004M), and 1:60 anti-Rpp2b (gift of Dr. Miguel Remacha). Anti-Rpp2b target recognition was confirmed by a specific 20 kDa upshift using a genomically TAP-tagged strain of *RPP2B* [108]. Membranes were washed and incubated with 1:10,000 HRP-conjugated goat anti-mouse IgG (Invitrogen 62–6520) or with 1:10,000 HRP-conjugated goat anti-rabbit IgG (Promega W4011) for 1 hour at room temperature. Secondary antibodies were detected with enhanced chemiluminescence and exposure to film.

### Quantification of western blots

To approximate the stoichiometry of Lso2 to ribosomes during stationary phase recovery, the Lso2-V5/Asc1 intensity ratio therein was compared to that of log phase. For each replicate, dilution series from both conditions were loaded on the same gel. Membranes from all conditions and replicates were exposed in parallel to the same piece of film. Band intensities were quantified in ImageJ. The slope of the best linear fit between either 3 or 4 consecutive points was used as the intensity of a single epitope. The Lso2-V5/Asc1 ratio of slopes was plotted for each growth condition. The same approach was used to quantify Lso2-V5/Asc1 ratios in +/− glucose (S1B Fig) and P-stalk protein abundance relative to Rpl8 (S7 Fig).

### Quantification of rRNA

Because saturated OD_600_ values were comparable between strains, we used total RNA in culture as a proxy for cellular abundance of RNA during stationary phase. Two mL of each culture was removed and pelleted. Total RNA was then extracted using hot acid phenol, precipitated, and resuspended in TE. From each sample, 2.5% was mixed with loading buffer (final concentrations of 50% formamide and 5% glycerol) for electrophoresis on synergel-agarose gels (0.9% synergel, 0.7% agarose, 1 μg/mL ethidium bromide, 0.5X TBE).

To measure the change in rRNA after 30 minutes of recovery from stationary phase, yeast strains were first grown for 96 hours to saturation and then diluted to OD_600_ 0.4 in fresh YPAD as described above. Thirty mL of culture was immediately removed and rapidly spun down. The cell pellet was flash frozen. After 30 minutes of shaking at 30°C, another 30 mL of the recovery culture was removed and spun down for flash freezing of the cell pellet. Because the OD_600_ is unchanged during this period, we used total RNA in culture as a proxy for cellular abundance of RNA. Total RNA extraction and analysis were performed as described above for stationary phase samples.

### Ribosome profiling library preparation

For ribosome profiling at 30 minutes of stationary phase recovery, 2 biological replicates of both *lso2Δ* and WT were grown. Each sample, which contained 2.0 L of culture, was harvested by rapid filtration (GE Healthcare 7184–009) and scraped into a conical tube. One mL of footprint lysis buffer was added (20 mM Tris-HCl pH 8.0, 140 mM KCl, 1.5 mM MgCl_2_, 1% Triton X-100, 0.1 mg/mL cycloheximide) and the cells immediately frozen in liquid nitrogen.

Library preparation followed the method of [65] with several modifications. Frozen cells were lysed by ball milling in a Retsch CryoMill. One minute of milling at 10 Hz followed by 1 minute of cooling was repeated 10 times. The lysate was spun for 5 minutes at 3,000 × *g* to remove debris. The supernatant was removed and further clarified for 10 minutes at 21,000 × *g*. The OD_260_ of each sample was determined by Nanodrop.

During analytical polysome profiling, we found that the contribution of ribosomes to total OD_260_ was highly variable between samples. However, dope-in of an internal standard required accurate quantification of total ribosome numbers. We therefore extracted total RNA from a fixed volume of each sample using hot acid phenol, followed by phenol-chloroform extraction in gel phase-lock tubes (Quantabio). At least 3 technical replicates of each sample were included to verify consistent recovery. Total RNA from all replicates was separated on a single 0.7% agarose/0.9% synergel (Diversified Biotech SYN100)/0.5X TBE gel containing 1.0 μg/mL ethidium bromide. A dilution series of total RNA mixed from several samples was also run on the same gel to ensure quantification in the linear range. The gel was scanned on a Typhoon FLA 9500. Using ImageJ, 18S and 25S band intensities were quantified. For each sample, the average of 18S + 25S intensities across technical replicates was used as the proxy for ribosome number per volume of lysate.

For RNase I digestion, lysate volumes adjusted to equalize total ribosome number between all samples contained anywhere from 18 to 38 OD_260_ units. The magnesium concentration was raised to 5 mM. RNase I was added to each sample at a concentration of 15 U/OD_260_, followed by incubation at 25°C for 60 minutes with gentle mixing. In parallel, HeLa lysate was footprinted with 0.5 U/μL RNase I at 25°C for 30 minutes; 0.74 OD_260_ units of digested HeLa lysate were added to each digested yeast sample. Mixtures were loaded onto 10%–50% sucrose gradients (20 mM Tris-HCl pH 8.0, 150 mM KCl, 5 mM MgCl_2_, 0.5 mM DTT, 0.1 mg/mL cycloheximide) and centrifuged at 35,000 rpm for 3 hours at 4°C in a Beckman SW41 rotor. Gradients were fractionated as described in “Yeast gradient profiling”; fractions from the 80S peak were pooled and extracted using hot acid phenol. Precipitated RNA was resuspended in 8 M guanidine HCl buffer (20 mM MES hydrate, 20 mM EDTA) and enriched for small RNAs as follows. Ethanol was added to 33% and the mixture spun through a Zymo-V column (Zymo Research C1012-50). The flow-through was collected and raised to 70% ethanol and then bound to a second Zymo-V column and eluted.

Footprints were dephosphorylated using T4 PNK (NEB M0201S) and then size-selected between 15 and 34 nt RNA markers on a 15% denaturing polyacrylamide gel. The pre-adenylated adapter OJA225 5′-5Phos/TGGAATTCTCGGGTGCCAAGG/3ddC/ was ligated to 3′ ends. Twenty picomoles or less of each sample was then treated with a half reaction of Ribo-Zero (Illumina MRZY1324). Samples were step annealed to the barcoded reverse transcription primer OWG921, followed by reverse transcription with AMV RT (Promega M5108). Gel-purified cDNA extension product was circularized with CircLigase (Epicentre CL4115K) and then PCR amplified with ONTI230 (forward) and OBC 5’-CAAGCAGAAGACGGCATACGAGATN_6_GTGACTGGAGTTCCTTGGCACCCGAGAATTCCA (reverse). Libraries were sequenced on a HiSeq 2500.

For ribosome profiling in log phase and at 3 hours of glucose withdrawal, 0.1 mg/mL cycloheximide was added to growing cultures and shaken for 2 minutes before harvesting. Downstream library preparation followed the method of [109], except that 2 additional subtractive hybridization oligos were used to deplete rRNA: 5′-/5Biosg/ATGACCAAGTTTGTCCAAATTCTCC and 5′-/5Biosg/AATGGGACCTTGAATGCTAGAACGTGG. Libraries were sequenced on a HiSeq 2000.

### Ribosome profiling read processing

For all libraries, reads were first demultiplexed by their library-level barcodes. For stationary phase recovery libraries, PCR duplicates were collapsed using fastx-collapser (FASTX-Toolkit) with the option <-Q33>. The output sequences were then trimmed of the 3′ adapter and 5′ decamer barcode using Cutadapt with the options <—adapter TGGAATTCTCGGGTGCCAAGG—cut 10—overlap 3—minimum-length 15>. Only reads ≥15 nt were retained.

For mapping of mixed human and yeast reads, the masked Sigma genome described in “eCLIP read processing and quantification” was concatenated with GRCh38 (GENCODE release 20) to build a joint reference. Reads were aligned uniquely using STAR with the command line options <—alignEndsType EndToEnd—outFilterMismatchNoverLmax 0.1—outFilterMultimapNmax 1—outFilterMultimapScoreRange 1—outFilterType BySJout—outFilterScoreMin 15>. Any reads that failed to align at this stage were excluded from further analysis. Aligned reads were then split by species.

Human reads were quantified using htseq-count [110] with the options <—format = bam—stranded = no—type = CDS—idattr = gene_id—mode = union>. These counts were converted to RPKMs; the length of a gene was taken to be the sum of exon lengths from all annotated isoforms.

Yeast reads were converted to genome coverage vectors of read 5′ ends. A read was assigned to a given ORF if its 5′ end was located between −12 of the start codon and −15 of the stop codon, inclusive. Footprint length histograms (Fig 6A) were determined from all alignments falling within annotated CDS regions. For DESeq2 analysis and calculation of RPKMs, all footprint lengths were used, but reads from the first 8 codons were excluded.

For analysis of [65,73], raw fastq data were taken from SRA SRX581790-2 and SRX2339595-8. The reference genome for mapping was a modified version of sacCer3 (R64-2-1_20150113) in which all noncoding RNAs at which 40 mers multimapped were masked from endogenous chromosomes. Single copies of all unique noncoding RNA genes were then placed on an artificial chromosome with intervening spacers. All other pipeline steps, including mapping, were identical to processing of Sigma reads.

Analysis of log phase and glucose starvation footprint libraries followed the method of [109], except that reads from the first 8 codons were excluded for expression quantification.

### Absolute normalization of yeast reads

To compare bulk numbers of mRNA-bound ribosomes between samples (Fig 4G), the number of uniquely mapped yeast CDS reads was divided by the number of uniquely mapped human CDS reads in each library. To rescale yeast RPKMs for comparison of individual genes, human RPKMs from *lso2Δ* replicate 1 (which had the highest human read coverage) were plotted against human RPKMs from the library to be normalized. Only the top 5% of human genes were used. The slope of the linear regression was applied as a global scaling factor to the yeast RPKMs in the library to be normalized.

### Ribosome profiling positional analyses

Metagene plots were created by dividing read counts at each position within a gene by the average read counts per nucleotide (expression level) of that gene. The values at each position were then averaged across the transcriptome. Only genes with ≥64 reads were used. Fig 5A was created with 28–30 mer footprints and genes ≥1,000 nt. S5A Fig was created with 20–22 mer footprints and genes ≥1,000 nt. Fig 6D was created with 28–30 mer footprints and genes ≥100 nt.

For all codon-level analyses, we found that 28 mers from ribosomes paused with the start codon in the P site had 5′ read ends located 12 nt upstream of the A of AUG, as previously observed [111] (Fig 5D). Other footprint lengths were therefore offset as follows to put them in frame with 28 mers (determined empirically): {20:0, 21:0, 22:+1, 25:0, 26:0, 28:0, 29:+1, 30:+1, 31:+1}. Twenty-seven mers were not well phased in our libraries. Reading frame analysis (Fig 6B) was performed on 20–22 mer 5′ read ends without offsets, in which “0” means that a read end is located a multiple of 3 from the first nucleotide of the start codon.

The ratio of start codon to body reads in Fig 5B was calculated using 25, 26, 28, 29, 30, and 31 mers. Start codon reads were taken as the number of offset 5′ read ends located 12 nt upstream of the A of AUG. For the same analysis of [65] (S5B Fig), all footprint lengths were used without offset because of their consistent 3′ phasing. Start codon reads were taken as the number of 3′ read ends located 15 nt downstream of the A of AUG. For analysis of start/body read correlation with DESeq changes (Fig 7C), 20–22 mers were also used in addition to long footprints. ORF body reads were taken as the total number of in-frame reads from the gene, excluding reads from the last 5 codons, and only genes with ≥64 reads were included.

The deviation of nucleotide usage in genes with start/body ratios sensitive to *lso2Δ* (Fig 5C) was calculated as follows. Genes were first sorted by their *lso2Δ*/WT change in start/body ratios, with ratios averaged between replicates. At each position *i*, the bias in usage of nucleotide *N* in the top quartile of genes was calculated as

$${bias}_{i}^{N}=2O_{i}^{N}ln(\frac{O_{i}^{N}}{E_{i}^{N}}),$$

where $O_{i}^{N}$ is the observed counts of *N* usage at position *i* in the top quartile, and $E_{i}^{N}$ is the expected counts. $E_{i}^{N}$ was computed by multiplying the total number of observations by the frequency of nucleotide *N* at position *i* in all genes analyzed. The overall G-statistic at position *i* was then calculated as

$$G_{i}=2\sum^{N}O_{i}^{N}ln(\frac{O_{i}^{N}}{E_{i}^{N}}).$$

The *p*-value was obtained from the chi-squared distribution with 3 degrees of freedom. *P*-values were then Bonferroni-adjusted to account for 26 simultaneous comparisons.

Individual codon pausing scores (Fig 5E–5G and S5C Fig) were calculated by the method of [111] using long footprints. For codon *i* at position 2 in gene *j*,

$$A\;site\;pause\;score(+2\;codon\;i,gene\;j)=\frac{\#read\;5^{\prime }\;ends\;corresponding\;to+2\;codon\;i\;in\;A\;site}{\frac{total\;\#\;in-frame\;reads\;in\;gene\;j}{\#\;codons\;with\;in-frame\;reads}}$$

The last 5 codons of each ORF were excluded from the denominator calculation. Pause scores from biological replicates were merged.

Aggregate metacodon occupancies (Fig 6C and S5D Fig) were calculated by the method described in detail by [111], which is based on the original method of [112]. In summary, the first nt of the P site was determined to be the 13th nt of a 28 mer based on ribosomes paused at the start codon. All other well-phased footprint lengths were shifted as described above to be in frame. For analysis of [73], we found that 20–22 mers required the same offsets as 20–22 mers from our own data. All genomic instances of a codon *NNN* were aligned. The number of ribosome footprints containing *NNN* in the E, P, and A sites and at flanking codons +1, +2, −1, and −2 were summed across the genome for each codon position. The occupancies for codon *NNN* at the E, P, and A sites were normalized to the averaged occupancy at +1, +2, −1, and −2. The first 8 and last 4 codons of each ORF were excluded, as well as any genes with an average coverage of <1 read per codon.

### Differential expression analysis

DESeq2 [79] was run on raw counts from all WT and *lso2Δ* footprint libraries, excluding counts from the first 8 codons of each ORF. Alpha was set to 0.05 for independent filtering; all other parameters were the DESeq2 defaults. *LSO2* itself was omitted from analyses of the decreased gene category. Gene Ontology *p*-values are Holm-Bonferroni corrected. Gcn4 targets were parsed from [80] as genes whose 2-fold induction during 3-aminotriazole treatment was dependent on *GCN4*.

### Software and statistical parameters

Analyses were performed with Python scripts or in Graphpad Prism 7 unless otherwise noted. Plots were made using Matplotlib, Graphpad Prism, and Integrated Genome Viewer. Sample sizes, technical and/or biological replicates, and the meaning of error bars are specified in figure legends. No analyses were done blinded.

## Supporting information

S1 Fig Related to Fig 1. (A) Lso2 is predicted to contain a coiled-coil domain, as well as 63 orthologs in 57 eukaryotes. A partial multiple sequence alignment is shown. (B) Cell extracts were prepared from +glucose and 2 hours −glucose. The same amount of total protein from each condition was loaded in a 2-fold dilution series and then probed for the V5 epitope and for Asc1 as a loading control. (Top) Representative western blot. (Bottom) Quantification of Lso2-V5 to Asc1 ratio, with the +glucose ratio normalized to 1. *n* = 3 biological replicates; mean ± S.D. (C) Log-phase extract from a Myc-tagged *LSO2* strain was fractionated through a sucrose gradient containing 25 mM EDTA. The indicated fractions were probed for the Myc epitope and for Asc1. The anti-Myc blots were exposed 5 times longer than those in Fig 1A. (D) A minority population of Sdd3 comigrates with ribosomes. Log phase or glucose-starved cell extract of a Myc-tagged *SDD3* strain was fractionated through a sucrose gradient. Each fraction was probed for the Myc epitope and for Asc1. (E) Lso1 expression is detectable during functional iron starvation. A Myc-tagged *LSO1* strain was grown during log phase with the indicated concentrations of copper ionophore or DMSO in combination with copper sulfate. Whole-cell extracts were probed for the Myc epitope and for Pgk1 as a loading control. Two-minute (short) and 15-minute (long) exposures of Myc-probing are shown. Pyrithione (upper) or neocuproine (lower) was used as the ionophore. Lso2, late-annotated short open reading frame 2; Pgk1, phosphoglycerate kinase 1; Sdd3, suppressor of degenerative death 3.
(TIF)

S2 Fig Related to Fig 2. (A) Myc-tagged Lso2 was grown to log phase with 4-thiouracil and crosslinked, as for all eCLIP experiments, before addition of cycloheximide to 0.1 mg/mL (Materials and methods). Cells were harvested and lysed for fractionation of extracts through a sucrose gradient. Fractions were probed for the Myc epitope and for Asc1. The asterisk denotes a cross-reacting species also present in Fig 1C. (B) (Left) Analysis of T-to-C transition frequency in rRNA-mapping reads. Only positions with read coverage ≥100 counts are shown. Shaded region indicates boundaries identified by eCLIP read coverage as IP specific (Fig 2B). The IP-specific crosslink is located at *RDN37* U4496 (25S U1253). Asterisks denote nonspecific species present in control libraries and correspond to positions *RDN37* 1206, 1557, 1604, 1884, 2377, 2892, 3763, 4673, 4849, 5240, 5452, and 6450. (Right) Inset of region containing the IP-specific crosslink. (C) The effect of RNase I concentration on the distribution of reads between tRNA versus rRNA features. Low, 1:2,000,000 dilution; high, 1:20,000,000 dilution. (D) Diagnostic electrophoresis membrane of radiolabeled Pus1-RNPs. Pus1 is a canonical tRNA modifying enzyme. Lane 1, IP; lane 2, untagged; lanes 3 and 4, western blot of Pus1-Myc in 0.5% of the input and in 13% of the IP, respectively. (E) Correlation of tRNA read densities between IP replicate 2 versus the SMI for ePAR-CLIP libraries made with 1:2,000,000 RNase I. Read density values were median centered and log_10_ transformed. (F) Coomassie staining of recombinant 6XHis-Lso2 purified from *E*. *coli*. Arrow indicates 6XHis-Lso2, which is 80% of the lane. eCLIP, enhanced crosslinking and immunoprecipitation; ePAR-CLIP, photoactivatable ribonucleoside crosslinking and immunoprecipitation and an enhanced method of CLIP library preparation; IP, immunoprecipitation; Lso2, late-annotated short open reading frame 2; Pus1, pseudouridine synthase 1; RNP, ribonucleoprotein; rRNA, ribosomal RNA; SMI, size-matched input.
(TIF)

S3 Fig Related to Fig 3. (A) HeLa cell extracts were fractionated through a sucrose gradient lacking magnesium and containing 2 mM EDTA. Western blots against CCDC124 were exposed 10 times longer than those in Fig 3B. (B) The yeast *LSO2* gene was swapped in a marker-free replacement with V5-tagged *CCDC124* containing the first 136 amino acids of the coding sequence, which is a putative shorter isoform of the gene [58]. (C) Diagnostic electrophoresis membrane of radiolabeled CCDC124-RNPs from ePAR-CLIP libraries prepared with 1:2,000,000 RNase I. Lanes 1 and 2, IP replicates; lane 3, untagged; lanes 4 and 5, western blot of CCDC124-Myc in 0.5% of the input and in 13% of the IP, respectively. The region from 55 kDa to 130 kDa was excised for each sample. The red line indicates the position of CCDC124-Myc alone (without crosslinked RNAs), based on the positions of protein markers. (D) As in S2B Fig. IP-specific crosslink is located at *RDN37* U4496 (25S U1253), as for Lso2. Asterisks denote nonspecific species present in control libraries and correspond to positions *RDN37* 848, 1206, 1485, 1515, 1544, 1577, 1604, 1884, 2377, 2892, 4108, 5128, and 6450. CCDC124, coiled-coil domain containing 124; ePAR-CLIP, photoactivatable ribonucleoside crosslinking and immunoprecipitation and an enhanced method of CLIP library preparation; IP, immunoprecipitation; Lso2, late-annotated short open reading frame 2; RNP, ribonucleoprotein.
(TIF)

S4 Fig Related to Fig 4. (A) WT and *lso2Δ* were grown to log phase in YPAD and then shifted to YPA (lacking glucose) for 3 hours before gradient profiling. *n* = 3 biological replicates; mean ± S.D. (B) The indicated strains were grown to mid-log phase and then plated in a 5-fold dilution series. Plates were imaged at 48 (30°C, 34°C, YPA+G, SC) or 72 hours (22°C, 37°C, YPA). Each plate contains 2 technical replicates. One of 2 biological replicates is shown. Temperature tests were done with YPAD plates. YPA+G, 2% glycerol as carbon source. (C) WT and *lso2Δ* were cultured in YPAD for 96 hours and then diluted to OD_600_ 0.1 in fresh medium to monitor outgrowth. (D) Correlations of RPKMs between biological replicates of WT (left) and *lso2Δ* ribosome footprint libraries (right), respectively, from stationary phase recovery. Genes with ≥64 reads in each library and the Pearson r^2^ are indicated. (E) Example of linear regression of human RPKMs between 2 libraries. *lso2Δ* replicate 1 (y-axis) was fixed as the normalizing library in all comparisons. R^2^ of linear fit is shown. The slope of the linear regression was applied as a global scaling factor to the yeast RPKMs in the library on the x-axis. Only human genes in the top 5% of RPKM values were used in each comparison. (F) Cell extracts from 30 minutes of stationary phase upshift were fractionated on a gradient containing 0.8 M KCl. Representative data from 2 biological replicates are shown. (G) (Left) Total RNA isolated from WT and *lso2Δ* strains recovering from 4 days in YPAD was separated by synergel-agarose electrophoresis. Time indicates minutes after switch to fresh medium. (Right) Quantification of 25S and 18S rRNA intensities at 0 and 30 minutes of recovery. For each strain, the rRNA intensity at 0 minutes was normalized to 1. *n* = 2 biological × 2 technical replicates; mean ± S.D. (H) (Left) Total RNA was isolated from equal culture volumes of WT and *lso2Δ* after 4 days of growth in YPAD. RNA from equivalent culture volumes was loaded in each lane and separated by synergel-agarose electrophoresis. Two biological replicates of each strain are shown. (Right) Quantification of 25S and 18S rRNA intensities in WT vs *lso2Δ*. *n* = 2 biological × 2 technical replicates; mean ± S.D. (I) RNase I–digested cell extracts from strains grown in YPAD for 4 days and then shifted to fresh medium for 30 minutes. Samples were first adjusted to contain equal numbers of ribosomes. Each sample was then treated with 15 U RNase I per OD_260_ unit for 60 minutes at 25°C, followed by fractionation through a sucrose gradient. Shown is a representative overlay from 3 biological replicates of each strain. RPKM, reads per kilobase per million reads; SC, synthetic complete; WT, wild type; YPA, yeast extract, peptone, adenine medium; YPA+G, YPA with glycerol; YPAD, YPA with glucose.
(TIF)

S5 Fig Related to Fig 5. (A) Metaribosome occupancy in a window around the start codon for 20–22 mer footprints. Numbers indicate the respective start codon occupancies in WT and *lso2Δ*. *n* = 2 biological replicates; mean ± S.D. (B) Histogram of change in the ratio of start codon to ORF body footprints for each gene with ≥64 reads in eIF5A depletion versus WT. Black trace (replicate error) indicates the same comparison for WT replicate 1 versus replicate 2. Data are from [65]. (C) Empirical cumulative distribution plots of the A site occupancy of each AGN codon when located at the +2 position. Single-codon pause scores from biological replicates were merged. (D) Comparison of A site codon occupancies for long footprints in *lso2Δ* versus WT ORF bodies. Biological replicates were averaged to compute the numerator and denominator, respectively. Error bars indicate propagated standard deviation of 2 biological replicates. eIF5A, eukaryotic translation initiation factor 5A; WT, wild type.
(TIF)

S6 Fig Related to Fig 7. Empirical cumulative distribution of the fold change in footprints for uAUG-containing versus all other genes. uAUG possession was determined from median 5′ UTR lengths in [113].
(TIF)

S7 Fig Related to discussion. WT and *lso2Δ* cells were grown to stationary phase for 4 days and then shifted to fresh medium for 30 minutes. Extracts were fractionated on sucrose gradients and the 80S fractions pooled. (Left) Representative western blots of the P-stalk proteins Rpp0 and Rpp2b. Rpl8 was used as an internal control for 60S loading. Each sample was loaded as a 2-fold dilution series. (Right) Quantification of western blots. *n* = 2 biological replicates and ≥1 technical replicate; mean ± S.D. WT, wild-type.
(TIF)

S1 Table Related to Fig 1. Proteins reproducibly identified by quantitative mass spectrometry of ribosomes from glucose-replete and glucose-starved conditions. Only proteins with ≥2 unique peptides between 2 biological replicates and ≥2 ratios in each biological replicate are included.
(XLSX)

S2 Table Related to Fig 2. Fold enrichments of tRNAs in Lso2 eCLIP IP samples relative to size-matched input or untagged controls, from set made with 1:2,000,000 RNase I. Only tRNA genes with ≥64 reads in all 4 libraries were analyzed. eCLIP, enhanced crosslinking and immunoprecipitation; IP, immunoprecipitation; Lso2, late-annotated short open reading frame 2.
(XLSX)

S3 Table Related to Fig 3. Fold enrichments of tRNAs in CCDC124 eCLIP IP relative to size-matched input or untagged controls. Only tRNA genes with ≥64 reads in all 4 libraries were analyzed. CCDC124, coiled-coil domain containing 124; eCLIP, enhanced crosslinking and immunoprecipitation; IP, immunoprecipitation.
(XLSX)

S4 Table Related to Fig 7. Full Gene Ontology annotations of differentially expressed genes in recovering *lso2Δ*. Terms with *p*-value of enrichment <0.05 (Holm-Bonferroni corrected) are shown.
(XLSX)

S5 Table Strains, primers, and synthetic gene constructs used for yeast genetics/cloning in this study. (XLSX)

## Abbreviations

6XHis-Lso2: recombinant His-tagged Lso2
Atf1/2: activating transcription factors 1/2
CCDC124: coiled-coil domain containing 124
CDS: coding sequence
Cld1: cardiolipin-specific deacylase 1
eEF1A: eukaryotic elongation factor 1A
eEF1Bγ: eukaryotic elongation factor 1Bγ
eEF2: eukaryotic elongation factor 2
eEF3: eukaryotic elongation factor 3
eIF5A: eukaryotic translation initiation factor 5A
eIF5A: eukaryotic translation initiation factor 5B
EM: electron microscopy
ePAR-CLIP: photoactivatable ribonucleoside crosslinking and immunoprecipitation and an enhanced method of CLIP library preparation
FDR: false discovery rate
GAC: GTPase activation center
Gcn4: general control nonderepressible protein 4
GFP: green fluorescent protein
HPF: hibernation promoting factor
IP: immunoprecipitation
K-S: Kolmogorov-Smirnov
LepA: Leader peptidase
Lso2: late-annotated short open reading frame 2
Lso2-Myc: yeast strains expressing Myc-tagged Lso2 at endogenous levels
MS/MS: tandem mass spectrometry
PDB: Protein Data Bank
PLB: Polysome Lysis Buffer
Pus1: pseudouridine synthase 1
RMF: ribosome modulating factor
RNP: ribonucleoprotein
RP: ribosomal protein
RPKM: read per kilobase per million reads
rRNA: ribosomal RNA
Sdd3: suppressor of degenerative death 3
SMI: size-matched input
Stm1: suppressor of Tom1
TEV: tobacco etch virus
WT: wild type
XIC: extracted ion chromatogram
