# Supplementary figures and images for: Lso2 is a conserved ribosome-bound protein required for translational recovery in yeast
Source: PLoS Biol. 2018 Sep 12;16(9):e2005903. doi: 10.1371/journal.pbio.2005903 (PMC6135351; doi:10.1371/journal.pbio.2005903)

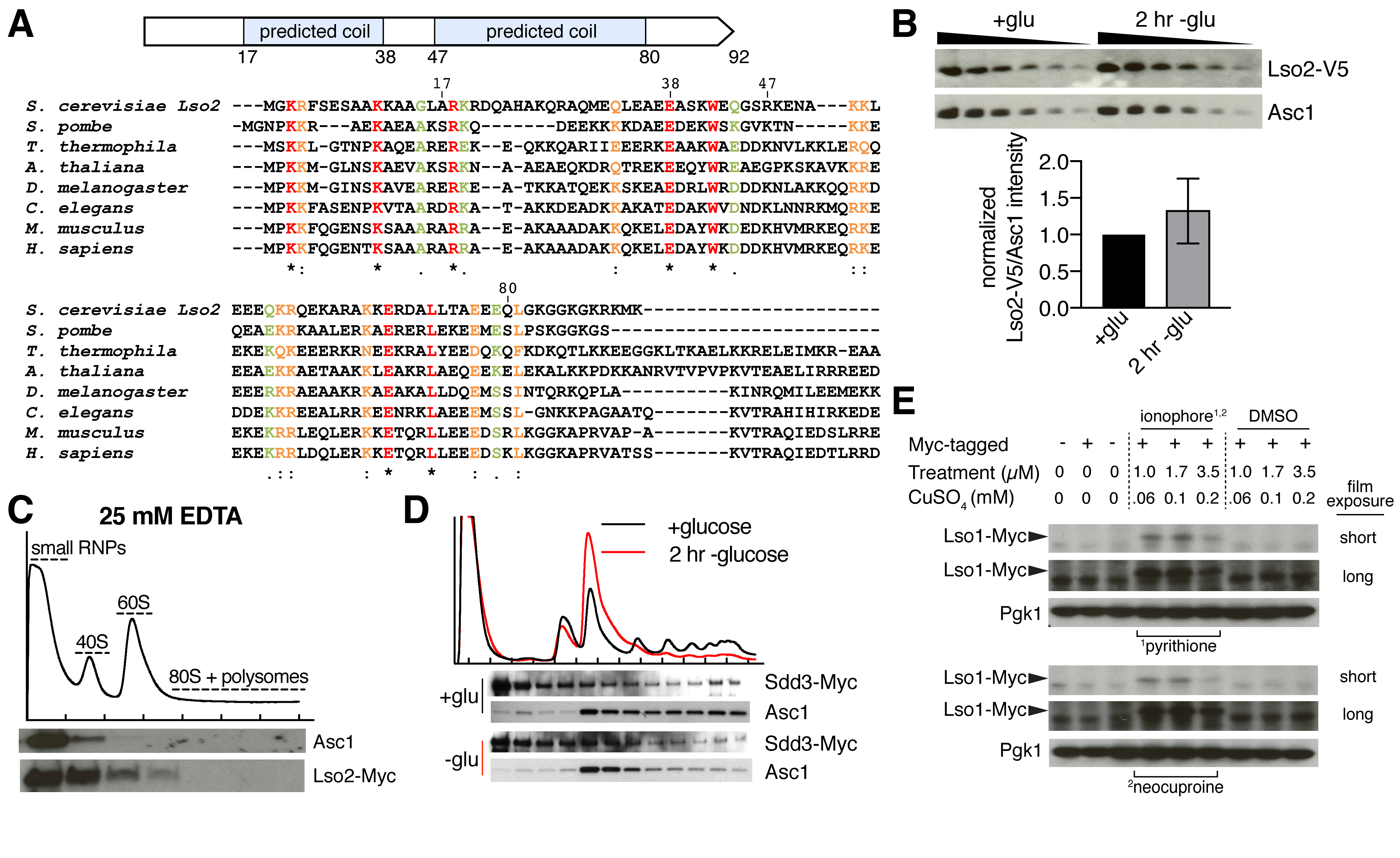

Supplement: S1 Fig — (A) Lso2 is predicted to contain a coiled-coil domain, as well as 63 orthologs in 57 eukaryotes. A partial multiple sequence alignment is shown. (B) Cell extracts were prepared from +glucose and 2 hours −glucose. The same amount of total protein from each condition was loaded in a 2-fold dilution series and then probed for the V5 epitope and for Asc1 as a loading control. (Top) Representative western blot. (Bottom) Quantification of Lso2-V5 to Asc1 ratio, with the +glucose ratio normalized to 1. n = 3 biological replicates; mean ± S.D. (C) Log-phase extract from a Myc-tagged LSO2 strain was fractionated through a sucrose gradient containing 25 mM EDTA. The indicated fractions were probed for the Myc epitope and for Asc1. The anti-Myc blots were exposed 5 times longer than those in Fig 1A. (D) A minority population of Sdd3 comigrates with ribosomes. Log phase or glucose-starved cell extract of a Myc-tagged SDD3 strain was fractionated through a sucrose gradient. Each fraction was probed for the Myc epitope and for Asc1. (E) Lso1 expression is detectable during functional iron starvation. A Myc-tagged LSO1 strain was grown during log phase with the indicated concentrations of copper ionophore or DMSO in combination with copper sulfate. Whole-cell extracts were probed for the Myc epitope and for Pgk1 as a loading control. Two-minute (short) and 15-minute (long) exposures of Myc-probing are shown. Pyrithione (upper) or neocuproine (lower) was used as the ionophore. Lso2, late-annotated short open reading frame 2; Pgk1, phosphoglycerate kinase 1; Sdd3, suppressor of degenerative death 3. (TIF) [file pbio.2005903.s001.tif]

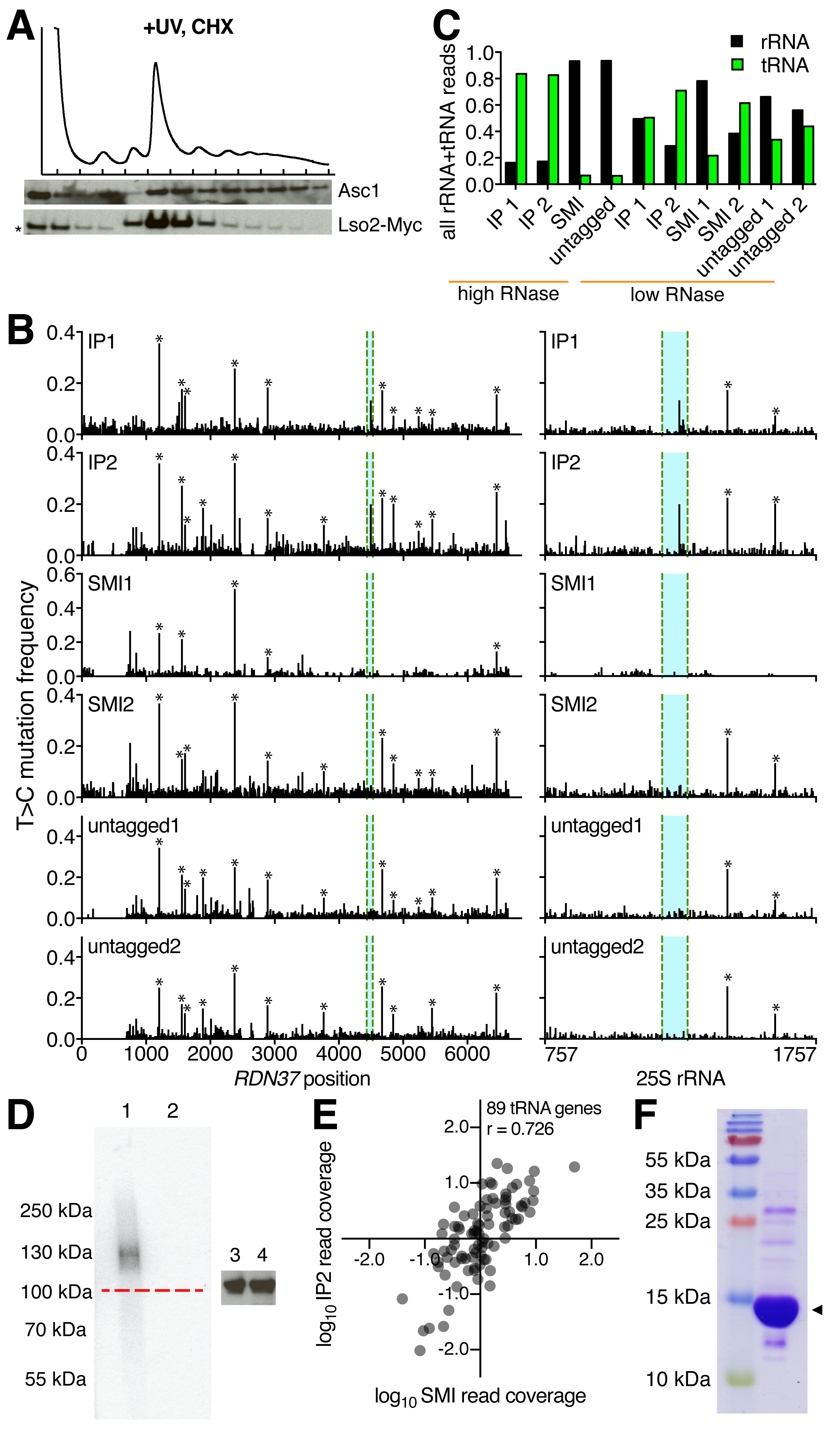

Supplement: S2 Fig — (A) Myc-tagged Lso2 was grown to log phase with 4-thiouracil and crosslinked, as for all eCLIP experiments, before addition of cycloheximide to 0.1 mg/mL (Materials and methods). Cells were harvested and lysed for fractionation of extracts through a sucrose gradient. Fractions were probed for the Myc epitope and for Asc1. The asterisk denotes a cross-reacting species also present in Fig 1C. (B) (Left) Analysis of T-to-C transition frequency in rRNA-mapping reads. Only positions with read coverage ≥100 counts are shown. Shaded region indicates boundaries identified by eCLIP read coverage as IP specific (Fig 2B). The IP-specific crosslink is located at RDN37 U4496 (25S U1253). Asterisks denote nonspecific species present in control libraries and correspond to positions RDN37 1206, 1557, 1604, 1884, 2377, 2892, 3763, 4673, 4849, 5240, 5452, and 6450. (Right) Inset of region containing the IP-specific crosslink. (C) The effect of RNase I concentration on the distribution of reads between tRNA versus rRNA features. Low, 1:2,000,000 dilution; high, 1:20,000,000 dilution. (D) Diagnostic electrophoresis membrane of radiolabeled Pus1-RNPs. Pus1 is a canonical tRNA modifying enzyme. Lane 1, IP; lane 2, untagged; lanes 3 and 4, western blot of Pus1-Myc in 0.5% of the input and in 13% of the IP, respectively. (E) Correlation of tRNA read densities between IP replicate 2 versus the SMI for ePAR-CLIP libraries made with 1:2,000,000 RNase I. Read density values were median centered and log10 transformed. (F) Coomassie staining of recombinant 6XHis-Lso2 purified from E. coli. Arrow indicates 6XHis-Lso2, which is 80% of the lane. eCLIP, enhanced crosslinking and immunoprecipitation; ePAR-CLIP, photoactivatable ribonucleoside crosslinking and immunoprecipitation and an enhanced method of CLIP library preparation; IP, immunoprecipitation; Lso2, late-annotated short open reading frame 2; Pus1, pseudouridine synthase 1; RNP, ribonucleoprotein; rRNA, ribosomal RNA; SMI, size-matched inpu [file pbio.2005903.s002.tif]

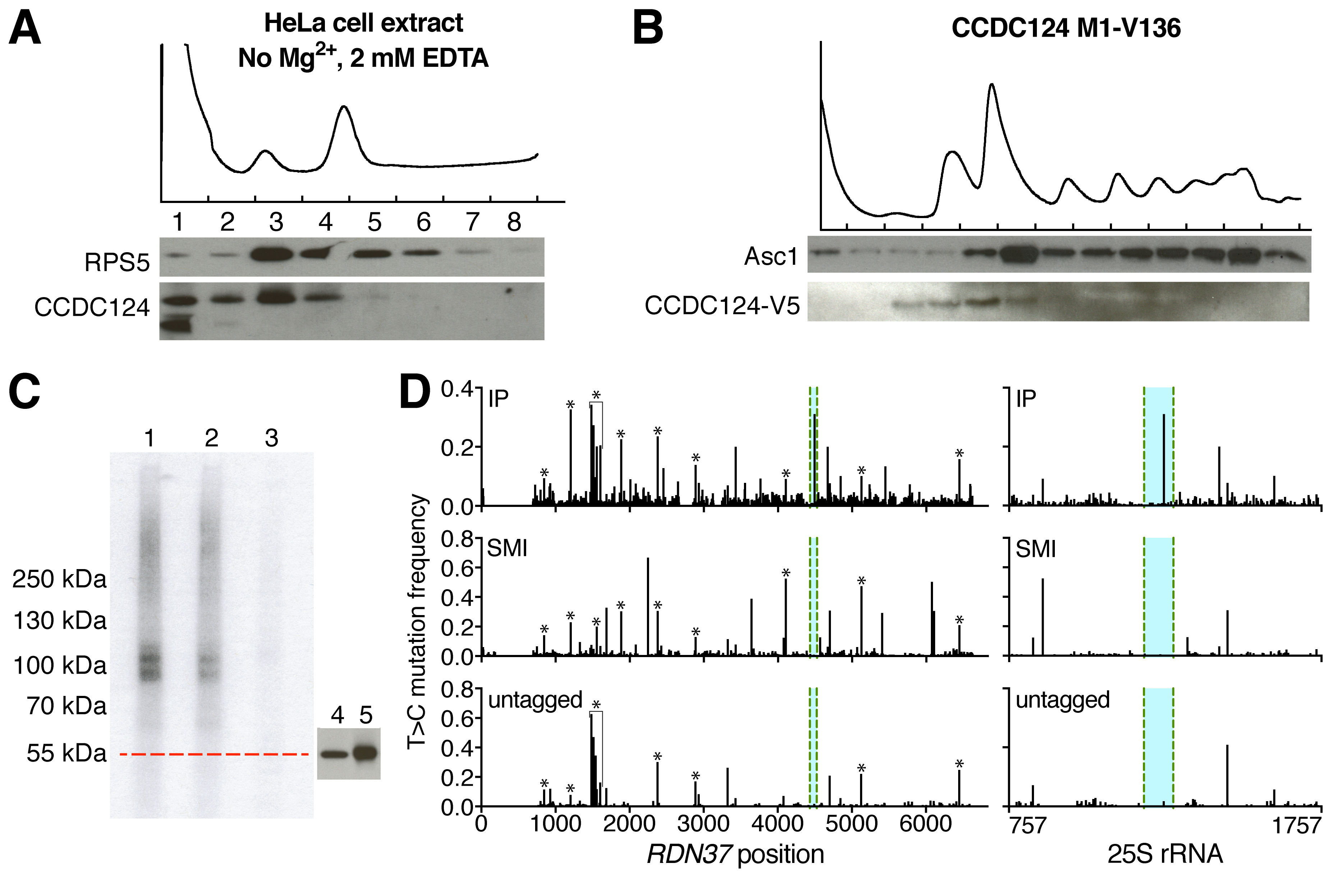

Supplement: S3 Fig — (A) HeLa cell extracts were fractionated through a sucrose gradient lacking magnesium and containing 2 mM EDTA. Western blots against CCDC124 were exposed 10 times longer than those in Fig 3B. (B) The yeast LSO2 gene was swapped in a marker-free replacement with V5-tagged CCDC124 containing the first 136 amino acids of the coding sequence, which is a putative shorter isoform of the gene [58]. (C) Diagnostic electrophoresis membrane of radiolabeled CCDC124-RNPs from ePAR-CLIP libraries prepared with 1:2,000,000 RNase I. Lanes 1 and 2, IP replicates; lane 3, untagged; lanes 4 and 5, western blot of CCDC124-Myc in 0.5% of the input and in 13% of the IP, respectively. The region from 55 kDa to 130 kDa was excised for each sample. The red line indicates the position of CCDC124-Myc alone (without crosslinked RNAs), based on the positions of protein markers. (D) As in S2B Fig. IP-specific crosslink is located at RDN37 U4496 (25S U1253), as for Lso2. Asterisks denote nonspecific species present in control libraries and correspond to positions RDN37 848, 1206, 1485, 1515, 1544, 1577, 1604, 1884, 2377, 2892, 4108, 5128, and 6450. CCDC124, coiled-coil domain containing 124; ePAR-CLIP, photoactivatable ribonucleoside crosslinking and immunoprecipitation and an enhanced method of CLIP library preparation; IP, immunoprecipitation; Lso2, late-annotated short open reading frame 2; RNP, ribonucleoprotein. (TIF) [file pbio.2005903.s003.tif]

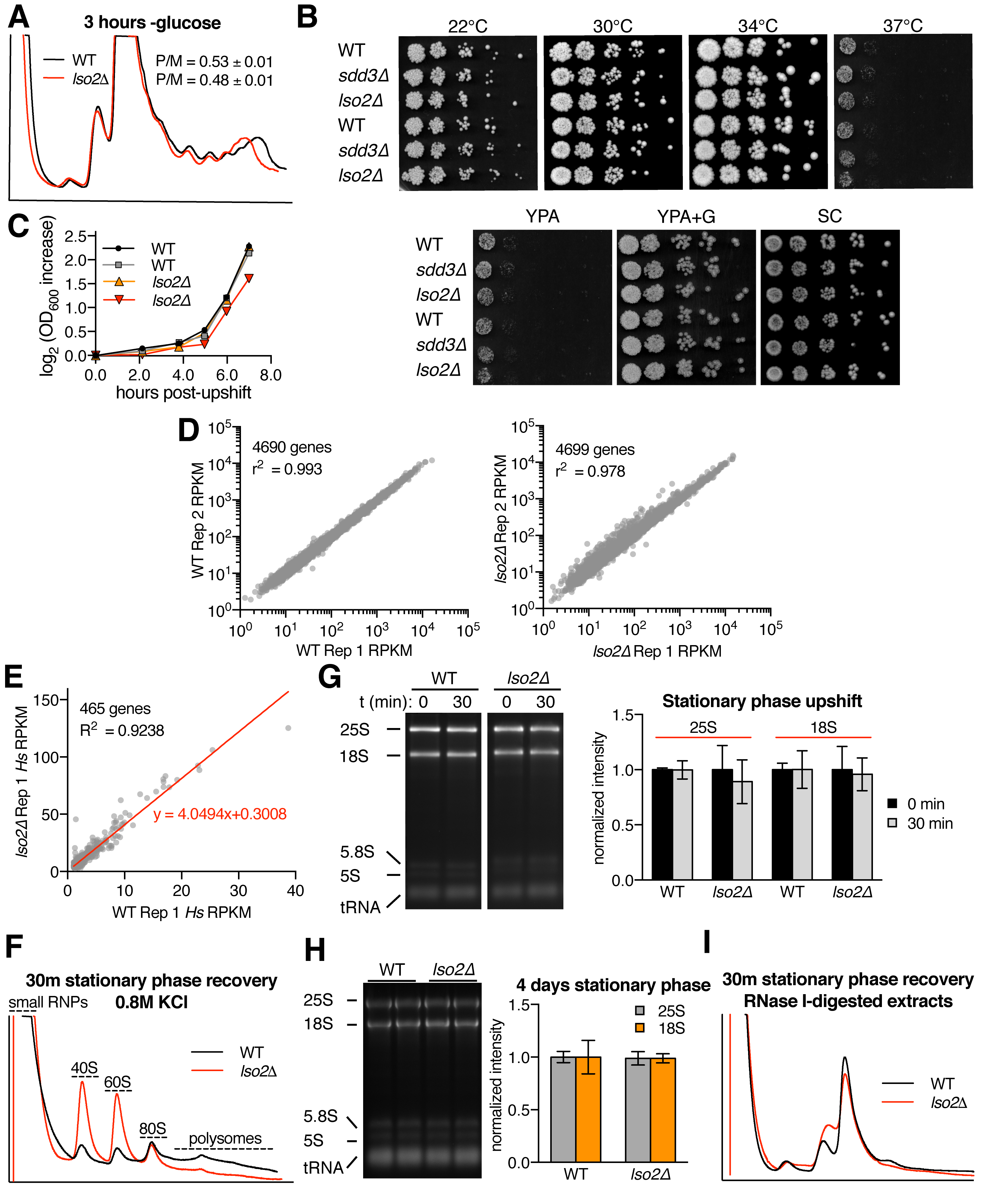

Supplement: S4 Fig — (A) WT and lso2Δ were grown to log phase in YPAD and then shifted to YPA (lacking glucose) for 3 hours before gradient profiling. n = 3 biological replicates; mean ± S.D. (B) The indicated strains were grown to mid-log phase and then plated in a 5-fold dilution series. Plates were imaged at 48 (30°C, 34°C, YPA+G, SC) or 72 hours (22°C, 37°C, YPA). Each plate contains 2 technical replicates. One of 2 biological replicates is shown. Temperature tests were done with YPAD plates. YPA+G, 2% glycerol as carbon source. (C) WT and lso2Δ were cultured in YPAD for 96 hours and then diluted to OD600 0.1 in fresh medium to monitor outgrowth. (D) Correlations of RPKMs between biological replicates of WT (left) and lso2Δ ribosome footprint libraries (right), respectively, from stationary phase recovery. Genes with ≥64 reads in each library and the Pearson r2 are indicated. (E) Example of linear regression of human RPKMs between 2 libraries. lso2Δ replicate 1 (y-axis) was fixed as the normalizing library in all comparisons. R2 of linear fit is shown. The slope of the linear regression was applied as a global scaling factor to the yeast RPKMs in the library on the x-axis. Only human genes in the top 5% of RPKM values were used in each comparison. (F) Cell extracts from 30 minutes of stationary phase upshift were fractionated on a gradient containing 0.8 M KCl. Representative data from 2 biological replicates are shown. (G) (Left) Total RNA isolated from WT and lso2Δ strains recovering from 4 days in YPAD was separated by synergel-agarose electrophoresis. Time indicates minutes after switch to fresh medium. (Right) Quantification of 25S and 18S rRNA intensities at 0 and 30 minutes of recovery. For each strain, the rRNA intensity at 0 minutes was normalized to 1. n = 2 biological × 2 technical replicates; mean ± S.D. (H) (Left) Total RNA was isolated from equal culture volumes of WT and lso2Δ after 4 days of growth in YPAD. RNA from equivalent culture volumes was loaded in each lane [file pbio.2005903.s004.tif]

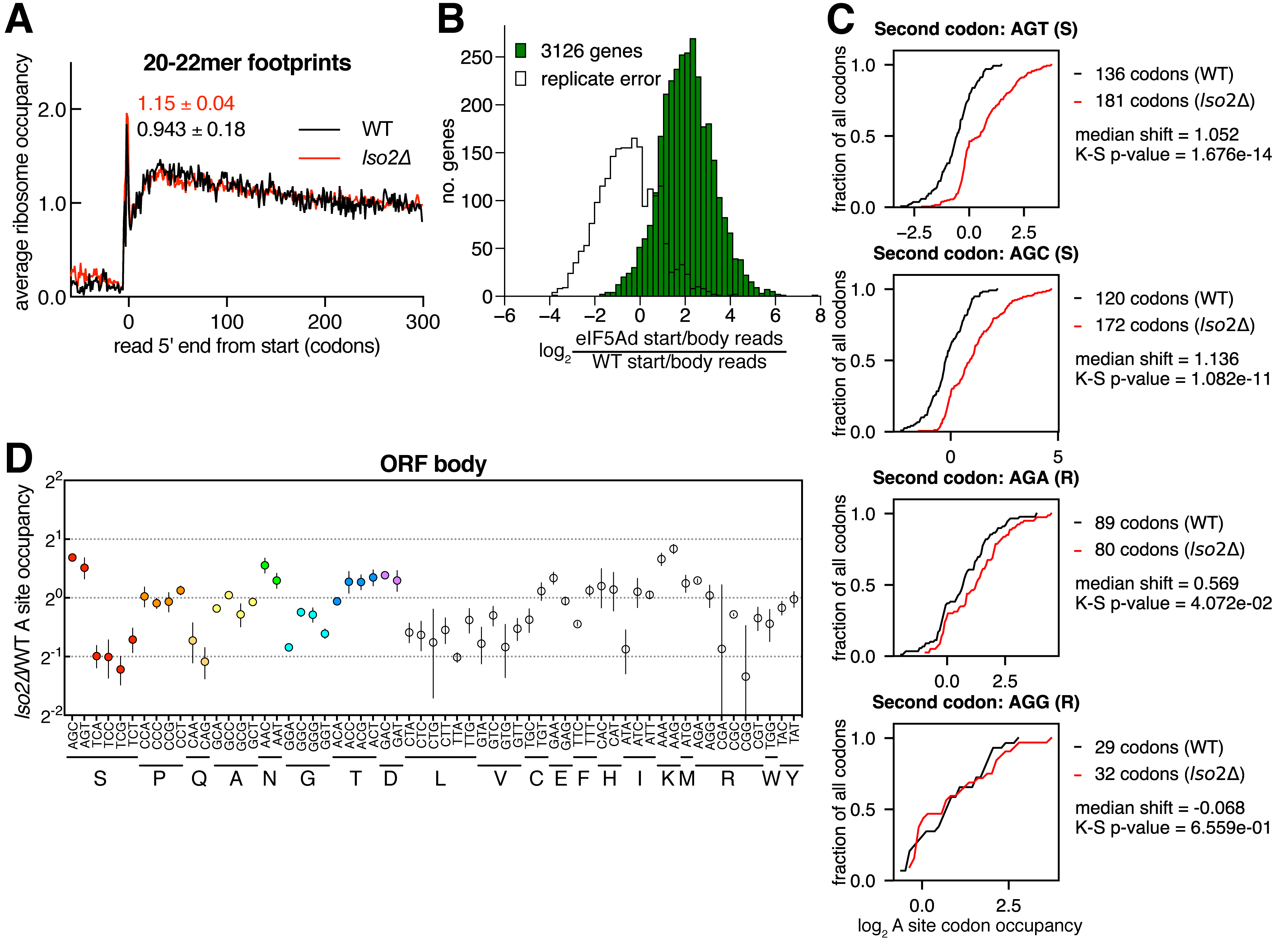

Supplement: S5 Fig — (A) Metaribosome occupancy in a window around the start codon for 20–22 mer footprints. Numbers indicate the respective start codon occupancies in WT and lso2Δ. n = 2 biological replicates; mean ± S.D. (B) Histogram of change in the ratio of start codon to ORF body footprints for each gene with ≥64 reads in eIF5A depletion versus WT. Black trace (replicate error) indicates the same comparison for WT replicate 1 versus replicate 2. Data are from [65]. (C) Empirical cumulative distribution plots of the A site occupancy of each AGN codon when located at the +2 position. Single-codon pause scores from biological replicates were merged. (D) Comparison of A site codon occupancies for long footprints in lso2Δ versus WT ORF bodies. Biological replicates were averaged to compute the numerator and denominator, respectively. Error bars indicate propagated standard deviation of 2 biological replicates. eIF5A, eukaryotic translation initiation factor 5A; WT, wild type. (TIF) [file pbio.2005903.s005.tif]

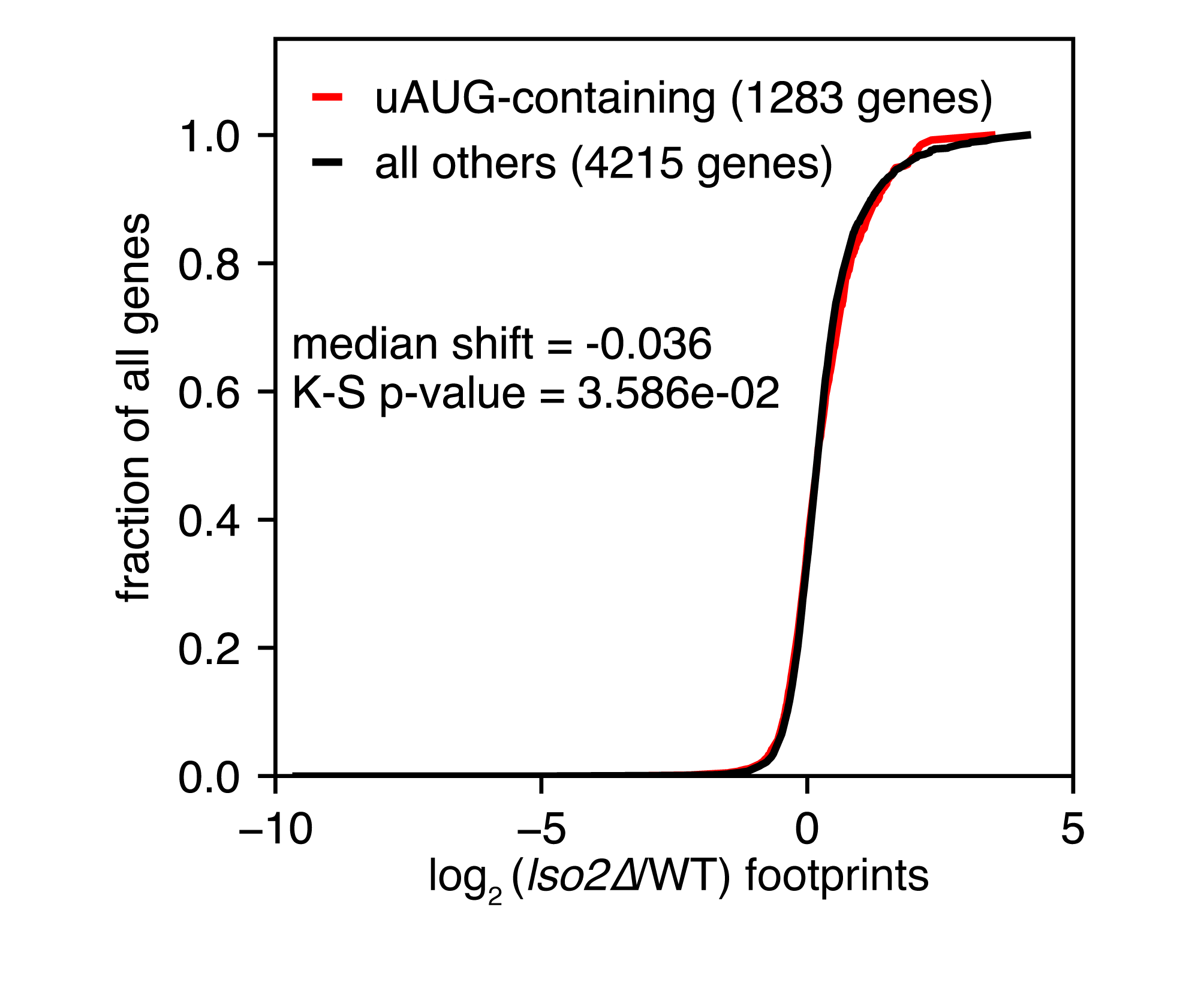

Supplement: S6 Fig — Empirical cumulative distribution of the fold change in footprints for uAUG-containing versus all other genes. uAUG possession was determined from median 5′ UTR lengths in [113]. (TIF) [file pbio.2005903.s006.tif]

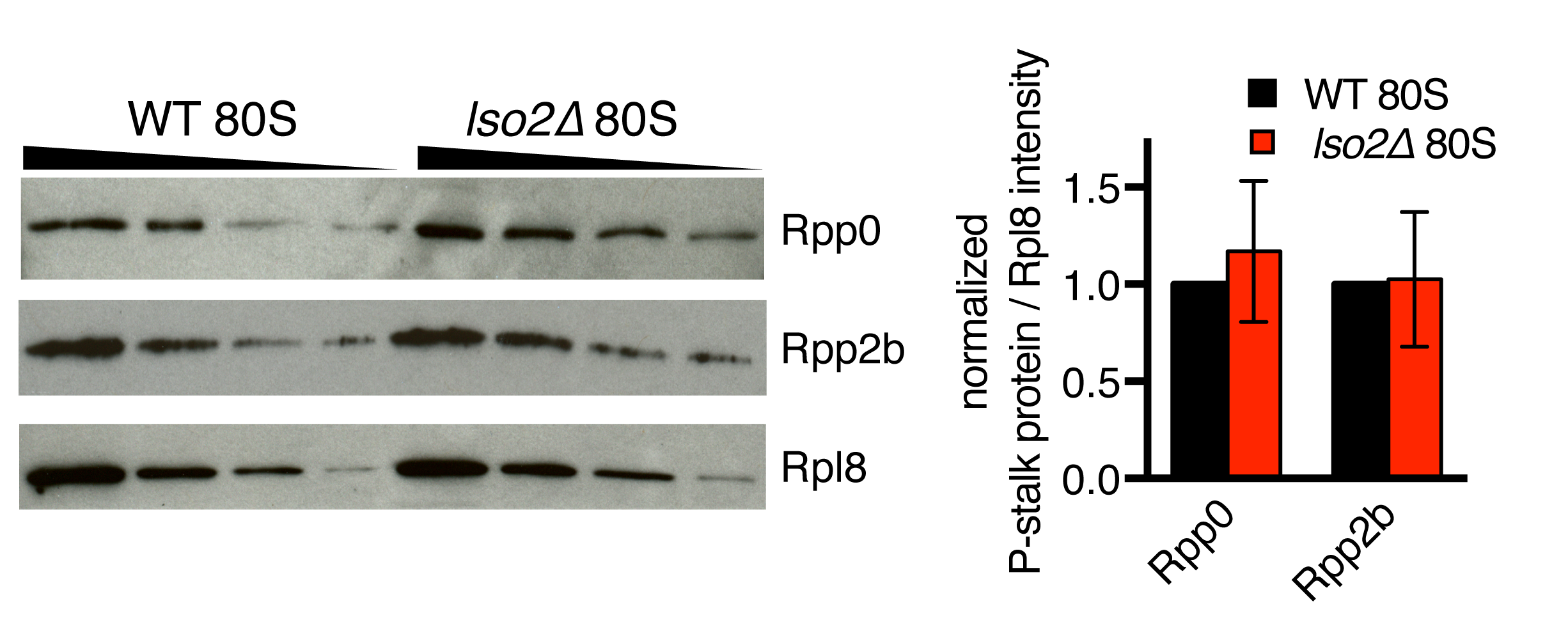

Supplement: S7 Fig — WT and lso2Δ cells were grown to stationary phase for 4 days and then shifted to fresh medium for 30 minutes. Extracts were fractionated on sucrose gradients and the 80S fractions pooled. (Left) Representative western blots of the P-stalk proteins Rpp0 and Rpp2b. Rpl8 was used as an internal control for 60S loading. Each sample was loaded as a 2-fold dilution series. (Right) Quantification of western blots. n = 2 biological replicates and ≥1 technical replicate; mean ± S.D. WT, wild-type. (TIF) [file pbio.2005903.s007.tif]
